# Cardiac ventricular Kir6.1 ATP-sensitive potassium channels: an overlooked effector of cardioprotection

**DOI:** 10.3389/fphys.2026.1808226

**Published:** 2026-04-20

**Authors:** Sean Brennan, Samir Makwana, Shen Chen, Lauren R. McGuinness, Moustafa Sweihi, Jacob A. Whitmore, Mahmoud E. Elghayesh, Christopher A. Martin, Noorann Sheikh, Manish Patel, Phoebe Malin, Mark W. Sims, Caroline Dart, Richard D. Rainbow

**Affiliations:** 1 Department of Cardiovascular and Metabolic Medicine & Liverpool Centre for Cardiovascular Sciences, University of Liverpool, Liverpool, United Kingdom; 2 Department of Cardiovascular Sciences, University of Leicester, Leicester, United Kingdom; 3 City University of New York School of Medicine, New York, NY, United States; 4 Department of Biochemistry, Cell and Systems Biology, University of Liverpool, Liverpool, United Kingdom

**Keywords:** ATP-sensitive potassium channel, cardiomyocytes, cardioprotection, KATP channels, Kir6.1

## Abstract

**Introduction:**

Adenosine triphosphate (ATP)-sensitive potassium (K_ATP_) channels are octameric structures, comprising a pore-forming homotetramer of Kir6.1 or Kir6.2, with 4 accessory sulphonylurea receptor (SUR) subunits. The canonical ventricular K_ATP_ channel is the highly ATP-sensitive Kir6.2/SUR2A complex, which is largely inactive under normal physiological conditions. Pharmacological activation of K_ATP_ channels is cardioprotective, but cardioprotective interventions, such as ischaemic preconditioning, preserve cellular ATP and cause a delay in Kir6.2/SUR2A activation. Recently we demonstrated functional expression of a second, Kir6.1-containing, ventricular K_ATP_ channel population that is constitutively active and modulates action potential duration. Here, we characterise the effects of cardioprotective stimuli on this newly identified K_ATP_ channel population.

**Methods:**

Patch-clamp recordings were used to investigate channel activity, in control cardiomyocytes, following adenosine or K_ATP_ modulator treatment, and in cardiomyocytes isolated from ischaemic-preconditioned whole hearts. Metabolic inhibition and washout experiments, together with whole-heart coronary ligation protocols, were used to assess markers of cardioprotection.

**Results:**

Cardioprotective stimuli increased Kir6.1 channel activity leading to action potential shortening, reduced Ca^2+^ accumulation and preserved contractile function, all hallmarks of a cardioprotected phenotype. Furthermore, inherent cardioprotection in female-derived cardiomyocytes correlates to increased Kir6.1 activity.

**Discussion:**

These findings suggest that the two functionally distinct populations of ventricular K_ATP_ channels play different roles in cardioprotection. Kir6.1-containing channels acutely control action potential duration, limiting Ca ^2+^ accumulation in the early stages of metabolic stress, whilst the canonical Kir6.2/SUR2A channel imparts late-stage protection against catastrophic ATP depletion. This role for Kir6.1 in cardioprotection suggests that this channel should be considered in drug development pipelines where channel block may inhibit endogenous cardioprotection.

## 1 Introduction

Adenosine triphosphate (ATP)-sensitive potassium (K_ATP_) channels were originally identified in cardiac muscle as ion channels that are inhibited by intracellular ATP (Noma, 1983). K_ATP_ channels have subsequently been identified in a number of different tissues where their biophysical and regulatory characteristics vary considerably, reflecting extensive molecular heterogeneity in their structure (reviewed by (Foster and Coetzee, 2016; Tinker et al., 2018)). Formed of homotetramers of either Kir6.1 or Kir6.2 pore-forming subunits, the channel cannot be expressed at the cell surface without the accessory sulphonylurea receptor (SUR)1 or SUR2 (of which there are A and B splice variants). The accepted view is that highly ATP-sensitive Kir6.2/SUR2A channels predominate in the heart (Nichols, 2016), although Kir6.1, Kir6.2, SUR1, SUR2A, and SUR2B have all been shown to be expressed at the sarcolemmal surface of ventricular cardiomyocytes (Singh et al., 2003; Morrissey et al., 2005a; Morrissey et al., 2005b; Aziz et al., 2014; Aziz et al., 2017; Aziz et al., 2018). Recently, we identified a second functional cardiac sarcolemmal K_ATP_ current formed from a Kir6.1 pore (Brennan et al., 2024). This Kir6.1-containing channel complex has a smaller conductance than the canonical Kir6.2/SUR2A complex; however, unlike Kir6.2/SUR2A, it is constitutively open in cardiomyocytes. This behaviour implies that it is not blocked to the same degree by ATP as its highly ATP-sensitive channel sibling, Kir6.2. Our previous study demonstrated that this Kir6.1-like current was inhibited by the Kir6.1 pore blocker PNU37883A, with a prolongation of the action potential (AP) (Brennan et al., 2024). The current was potentiated maximally by 10 µM pinacidil, unlike the canonical Kir6.2/SUR2A cardiac K_ATP_ complex, which required 150–200 µM to fully activate, with a pinacidil concentration dependence on the AP shortening (Brennan et al., 2024). Finally, the Kir6.1-like single-channel activity was markedly reduced in a shRNA *KCNJ8* knockdown approach in isolated cardiomyocytes, whereas the single-channel activity was absent in a *KCNJ8^−/−^* knockout mouse model (Brennan et al., 2024).

Although predominantly inactive under normal physiological conditions due to ATP inhibition (Inagaki et al., 1995; Inagaki et al., 1996), ventricular Kir6.2/SUR2A channels open during severe metabolic stress, such as ischaemia (Noma, 1983), causing shortening of the AP, reduction of Ca^2+^ entry, and contractile activity, as crucial energy-sparing protective mechanisms (Cole et al., 1991; Suzuki et al., 2002; Nichols, 2016). Intrinsic cardioprotective stimuli, such as ischaemic preconditioning (IPC), or the pharmacological activators of K_ATP_ channels, also lead to AP shortening under physiological conditions via activation of K^+^ conductance(s) (Schulz et al., 1994; Brennan et al., 2015). This is unlikely to be due to Kir6.2/SUR2A channel activation, as cardioprotective stimuli preserve intracellular (ATP) levels (Kaplan et al., 1994), and indeed, the opening of Kir6.2/SUR2A channels is markedly *delayed* during simulated ischaemia following IPC or pretreatment with adenosine (Brennan et al., 2015). We therefore hypothesised that populations of sarcolemmal cardiac Kir6.1-containing K_ATP_ channels, distinct from the canonical Kir6.2/SUR2A, may play an important protective role.

Here, we present data that show that in cardiomyocytes, cardioprotective stimuli (e.g., adenosine or IPC) or pharmacological agents increase the constitutive activity of Kir6.1-containing channels, leading to a modest but significant shortening of AP duration (APD). This reduces Ca^2+^ accumulation during each contractile cycle, which lowers ATP consumption during periods of metabolic compromise. This fine-tuning of APD helps preserve cellular ATP levels to ensure that Ca^2+^ homeostasis is maintained and protects against the cytosolic Ca^2+^ build-up that is so damaging in reperfusion injury. Kir6.2/SUR2A, on the other hand, acts as the last line of defence against complete ATP depletion by passing a large hyperpolarising current that shuts down contraction to minimise further ATP consumption in its role as an ATP sensor.

## 2 Methods

### 2.1 Animals

Whole hearts or cardiomyocytes isolated from adult male or female Wistar rats (200–400 g) were used in this study. Adult male and female Wistar rats have been widely used in the literature as a source of ventricular myocytes for mechanistic and pharmacological studies of myocardial function. All rats used in this study were purchased from Charles River Laboratories (Margate, UK) and housed in the animal unit at the University of Leicester or Liverpool. Animals were provided when they reached an appropriate size (over 200 g), and the researchers had no input into the selection of the animals. Experiments were not blinded in this study, as the experimenter was responsible on each day for the cell preparation, solution making, and analysis of each completed dataset. To reduce any experimental bias or anomalous results due to errors in experiments, solutions, or differences in cellular behaviour from an animal, all data are expressed as *n* = animal. Multiple experimental protocols were carried out on each day to ensure the best use of each cell preparation. For whole-cell or action potential recordings, data were recorded from at least five animals in each case. For contractile function and calcium fluorescence measurements, fields of view were recorded from cells isolated from at least five animals, and the mean of the animals was used in all cases. For coronary ligation experiments, a single user carried out the experimental protocol and analysed the recording. A second user also analysed the data, and the mean data analysis from both users was used for the figures.

### 2.2 Ethical statement

Adult male or female Wistar rats were killed by concussion and cervical dislocation. The care and schedule 1 killing of animals conformed to the requirements of the United Kingdom Animals (Scientific Procedures) Act 1986 Amendment Regulations (SI 2012/3039). Ethical approval for all experimental procedures was granted by the University of Leicester/Liverpool’s Animal Welfare and Ethical Review Body (AWERB_2018_44 (Leicester) or AWC0152-AWERB (Liverpool)). The ARRIVE 2.0 guidelines for reporting experiments involving animals were followed (Lilley et al., 2020; Percie du Sert et al., 2020).

### 2.3 Sex as an experimental variable

The effect of sex and hormones on cardiovascular function is an area of ongoing research (Chicco et al., 2007; Blenck et al., 2016). Female animals display inherent cardioprotection that is presumably due to different sex hormone levels (Jovanovic et al., 2000; Ma et al., 2009; Murphy et al., 2011; Bendale et al., 2013). In this study, we are comparing cardioprotected vs. control cells, and therefore, we have used predominantly male animals, as we cannot isolate noncardioprotected female cells. To assess whether the cardioprotection in female animals occurs via the mechanisms proposed in this study, female animals have also been used in some confirmatory experiments.

### 2.4 Isolation of ventricular myocytes

Adult male or female Wistar rats (200–400 g) were killed by the Schedule 1 procedure of concussion and cervical dislocation. Following schedule 1, the heart was quickly removed from the thoracic cavity and briefly submerged in cold (4 °C) Ca^2+^-free Tyrode’s solution containing: 5 mM KCl, 135 mM NaCl, 0.33 mM NaH_2_PO_4_, 5 mM Na pyruvate, 10 mM HEPES, 15 mM mannitol, 5 mM glucose, 1 mM MgCl_2_, and 0.3 mM EGTA, pH 7.4 (all chemicals were from Merck, Gillingham, UK), to halt contractions and lower the metabolic demand. The isolated heart was then cannulated via the aorta, mounted on a Langendorff-type apparatus, and perfused in a retrograde manner with warmed Ca^2+^-free Tyrode’s solution (37 °C) for 6 min. The enzyme mix solution (0.8 mg/ml collagenase; 1.66 mg/ml BSA prepared from factor V albumin, and 0.5 mg/ml protease; type XIV, 15% Ca^2+^; Merck, Gillingham, UK) was then perfused through the heart until isolated cells appeared in the perfusate sampled from the ventricles. The solution was then exchanged for Ca^2+^-free Tyrode’s solution for a further 2 min, and then the heart was cut down, washed in normal Tyrode’s (NT) solution containing: 5 mM KCl, 135 mM NaCl, 0.33 mM NaH_2_PO_4_, 5 mM Na pyruvate, 10 mM HEPES, 15 mM mannitol, 5 mM glucose, 1 mM MgCl_2_, 2 mM CaCl_2_, and cardiomyocytes mechanically separated using a shaking water bath at 37 °C. This technique yielded 70%–90% rod-shaped cardiomyocytes, which were stored in NT solution at room temperature and used within 12 h of isolation.

### 2.5 *Ex vivo* whole-heart ischaemic preconditioning protocol

Following schedule 1 killing, hearts were cannulated on the Langendorff apparatus and perfused retrogradely for 10 min in warmed Tyrode’s solution (37 °C). The solution was then exchanged for a pyruvate-free Tyrode’s solution for a further 5 min. The heart was then subjected to three cycles of 5 min of halted perfusion (global ischaemia) and 5 min of washout with pyruvate-free Tyrode’s solution. On the final reperfusion, the pyruvate-free Tyrode’s solution was exchanged for our standard Tyrode’s solution (for whole-heart *ex vivo* coronary ligation experiments) or for a nominally calcium-free Tyrode’s solution for isolated ischaemic preconditioned cardiomyocytes. This protocol has been routinely used in our group (Sims et al., 2014; Brennan et al., 2015).

### 2.6 *Ex vivo* coronary ligation experiments

Following schedule 1 killing by concussion and cervical dislocation, hearts from adult male or female Wistar rats (200–400 g) were quickly removed and cannulated on a Langendorff apparatus and perfused retrogradely with warmed Tyrode’s solution (37 °C) for 1 h to stabilise, or underwent the IPC protocol outlined above. The left anterior descending coronary artery was then ligated for 40 min to cause ischaemia, using 5–0 USP braided silk suture and two pipette tips to form a reversible knot around the artery. The knot was then removed to start the 3-h reperfusion phase, with the suture remaining in place to allow for re-ligation. During all phases, temperature was carefully maintained at 37 °C by submerging the heart in NT solution using a heated water jacket. Evans Blue dye (1% in Tyrode’s solution) and 2,3,5-triphenyltetrazolium chloride (Sigma-Aldrich, Merck, Gillingham, UK) were used to identify the area at risk (AAR) and infarcted area (IA), respectively. To determine the AAR and IA, each slice was scanned on both sides and weighed. AAR and IA were calculated for each of the slices from the heart using ImageJ. The AAR, IA, and unaffected area sizes from ImageJ were then used to calculate the percentage infarct of the AAR by weight (Brennan et al., 2019; Brennan et al., 2022; Brennan et al., 2023). For drug-treated coronary ligation experiments, the drug was perfused for 5 min prior to ligation and remained present in the solution until reperfusion, 40 min following ligation.

### 2.7 Patch-clamp electrophysiology

Patch electrodes were made from filamented thick-walled borosilicate glass with a resistance of 3–6 MΩ. Recordings were made from isolated cardiomyocytes using an Axopatch 200B amplifier, with filtering at 2 kHz. Recordings were digitised using a Digidata 1440 and recorded and analysed using pCLAMP 11.3 software (Axon Instruments, Scientifica, Uckfield, UK, RRID: SCR_011323). Cells were allowed to adhere to a glass coverslip mounted in a Dagan HW-30 heated perfusion chamber (Dagan Corp, Minneapolis, MN, USA) for 10 min prior to experimentation. Solutions were perfused at a rate of 5 ml/min and at 32 °C ± 2 °C.

#### 2.7.1 Cell-attached

Intracellular electrode solution contained 140 mM KCl, 10 mM 4-(2-hydroxyethyl)-1-piperazineethanesulfonicacid (HEPES), 1 mM CaCl_2_, and 0.5 mM MgCl_2_, pH 7.4 with KOH. In voltage-clamp mode, the electrode was held at + 40 mV for the duration of the recording. With these solutions, the equilibrium potential for potassium is close to 0 mV, and the voltage across the membrane patch is approximately − 110 mV (the sum of the electrode potential and the resting potential of the cardiomyocytes, assumed to be − 70 mV), so that single K_ATP_ channel openings lead to inward currents.

Kir6.1 channel activity was identified by its single-channel current amplitude at − 110 mV (the approximate membrane potential across the cell-attached patch). In our previous manuscript, we demonstrated that this channel was present with a conductance of ~ 40 pS, consistent with the literature regarding the conductance of this channel, and showed constitutive activity (Brennan et al., 2024). At − 110 mV, the single-channel current from Kir6.1 would be ~ 5.5–6 pA, thus making it distinct from the IK_1_ current (Kir2.x channels), with a single-channel current amplitude of ~ 3–3.5 pA. At − 110 mV, IK_1_ (Kir2.x channels) would be expected to be the primary other active ion channel, with all voltage-gated channels held in a very low open-probability state. No non-K_ATP_ channel blockers were applied as part of our routine investigations, as Kir6.1 and Kir2.x channels could be readily distinguished by their amplitude and their biophysical characteristics. Kir6.1 demonstrates bursting opening behaviour whilst Kir2.x opens for long durations that could be treated like a change in baseline in the single-channel analysis (examples of this are shown in our previous manuscript) (Brennan et al., 2024).

To investigate channel activity, the channel open probability (*P*_O_) was calculated; however, in the majority of recordings, multiple levels of opening were observed, and therefore, the number of levels and the duration of activity at each level were measured:


$$T_{\text{O}}=\sum_{L=1}^{N}t_{\text{oL}}$$

*T*_O_ represents the duration that the channel was open, where *L* represents different open levels, *t*_oL_ is the duration of time in each level, and *N* is the number of ion channels in the recording. Given that the true number of channels cannot be measured because of the stochastic behaviour of ion channels, the number of channels is taken as the maximum number of levels open during the total (*T*) duration of the recording. The open probability is presented as NP_O_:


$${\text{NP}}_{\text{O}}=\frac{T_{\text{O}}}{T}$$

**Figure 1:**
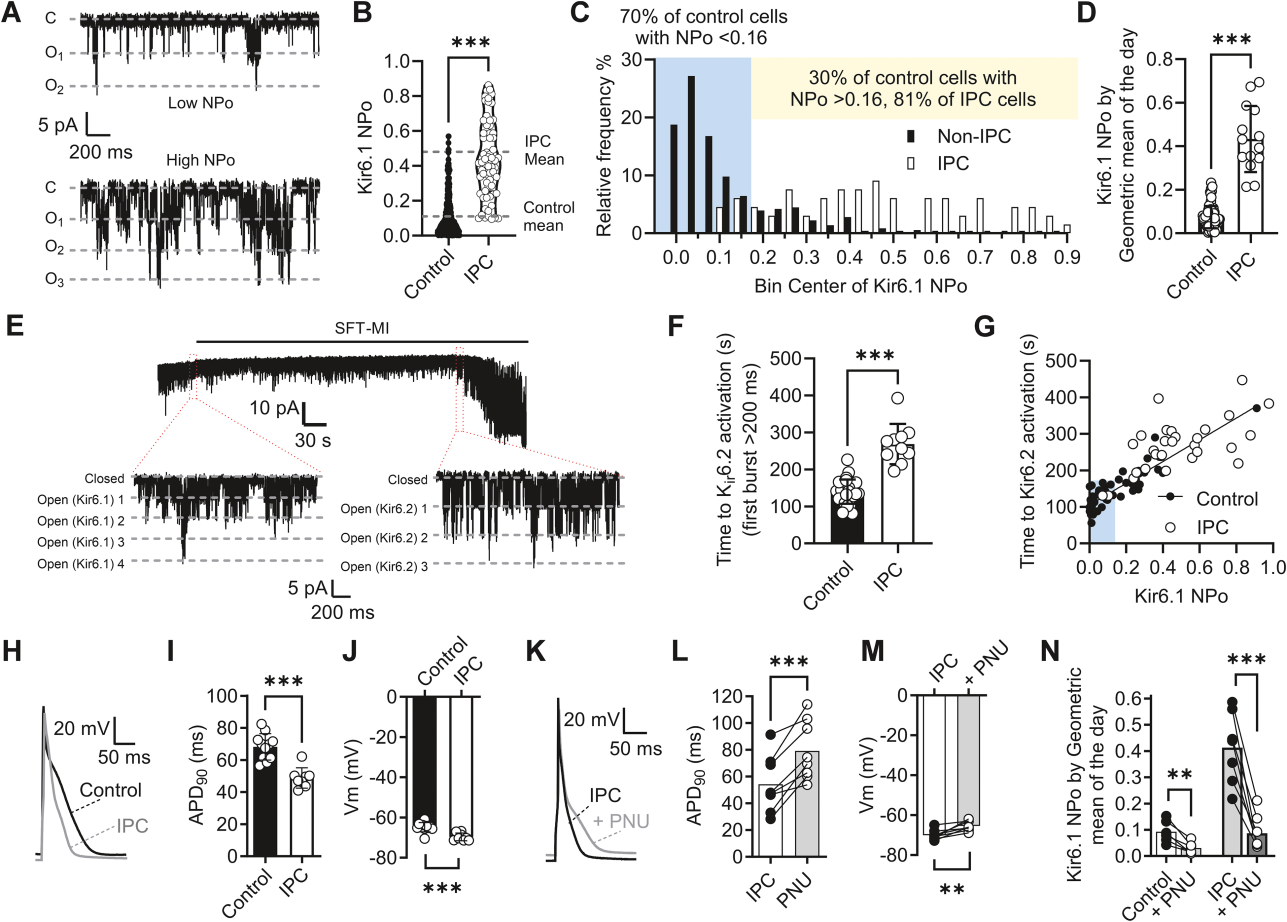
Kir6.1 activity in ventricular cardiomyocytes is increased following a whole heart ischaemic preconditioning protocol. **(A)** Example traces of Kir6.1 from a control cell with a low NP_O_ and one with a high NP_O_. **(B)** Mean NP_O_ from 357 control (0.11 ± 0.10, 73 animals) and 66 IPC cells (0.44 ± 0.23, 14 animals). ^***^p < 0.001. Data were not normally distributed, and therefore a nonparametric Mann–Whitney *U* test was used. **(C)** Distribution histogram of NP_O_ for IPC and control cells in 0.05 bins. In control cardiomyocytes, 70% of cells had an NP_O_ < 0.16, whereas 30% had an NP_O_ > 0.16. In IPC cardiomyocytes, this was shifted to 81% of cells having an NP_O_ > 0.16. **(D)** Mean Kir6.1 NP_O_ expressed as the geometric mean of the day. ^***^p < 0.001. Data were not normally distributed; therefore, a nonparametric Mann–Whitney *U* test was used (*n* = 73 and 12 animals for control and IPC hearts, respectively). **(E)** Example trace of a cell-attached patch recording from an IPC cardiomyocyte showing the opening of Kir6.2/SUR2A following perfusion with SFT-MI solution. Expanded traces show Kir6.1 openings prior to, and Kir6.2/SUR2A openings during, SFT-MI perfusion. **(F)** Mean time to Kir6.2/SUR2A channel opening in control and IPC cells, showing a significant delay in the time to Kir6.2/SUR2A opening (^***^p < 0.001, unpaired *t*-test, *n* = 26 and 10 animals in the control and IPC groups, respectively). **(G)** Time to Kir6.2/SUR2A opening during SFT-MI perfusion plotted against the Kir6.1 NP_O_ of that cell (*n* = 57 (26 animals) and 29 (10 animals) control vs. IPC cells, respectively. **(H)** Example action potentials from control and IPC cardiomyocytes. Mean APD_90_ (^***^p < 0.001) **(I)** and Vm (^***^p = 0.001) **(J)** from control and IPC cells were analysed using an unpaired *t*-test, with *n* = 11 (Esposito et al., 2025) and 8 (Bendale et al., 2013) animals **(cells)** for control and IPC cardiomyocytes, respectively. **(K)** Example action potential traces from an IPC cardiomyocyte in the absence and presence of 3 µM PNU37883A. Mean APD_90_ (^***^p < 0.001) **(L)** and Vm (^**^p = 0.004) **(M)** from IPC cardiomyocytes in the absence and presence of 3 µM PNU37883A were analysed using a paired *t*-test (*n* = 8 [12] animals [cells]). **(N)** Mean NP_O_ in the absence and presence of 3 µM PNU37883A in control and IPC cardiomyocytes (^**^p = 0.004; ^***^p < 0.001, *n* = 6 [9] and 7 [12] animals [cells]). Data were analysed using repeated-measures two-way ANOVA with uncorrected Fisher’s posttest.

Sampling rate for cell-attached recordings was 50 kHz (Brennan et al., 2015; Brennan et al., 2024). Data were analysed in pCLAMP10 using a threshold set to 4.5 pA per open channel level. This allowed the 30-pS Kir2.x current to be largely filtered out as a change in baseline, given its long open durations, compared with the larger (40 pS), bursting Kir6.1-like channel (Brennan et al., 2024). Given the variability of the NP_O_, as reported in **Figure 1**, the geometric mean of the day was used for recordings in the cell-attached configuration. Data have been presented as both individual cells and as the geometric mean of the day, where data are, therefore, reported as *n* = animals. Sixty seconds of data were used to measure the NP_O_ for each cell. Data are expressed as paired (where control recordings at the end of 3 min of recording are then compared to the end of 4 min of perfusion with the drug) or unpaired (when separate recordings are made on the day for control and drug-treated cells). In all cases, a single cell-attached patch recording was made per cell.

#### 2.7.2 Whole-cell recording

Intracellular electrode solution contained 30 mM KOH, 110 mM KCl, 10 mM EGTA, 10 mM HEPES, 1 mM MgCl_2_, 1 mM Mg-ATP, 0.1 mM Na-ADP, 0.1 mM GTP, and 0.61 mM (20 nM free) CaCl_2_, pH 7.2 with HCl.

#### 2.7.3 Action potential recording

In current-clamp mode, APs were stimulated at 1 Hz via the patch electrode with a 5-ms depolarising trigger, set to 130% of that required to elicit an AP (approximately 500–900 pA). Action potential duration to 90% repolarised (APD_90_) and membrane potential (*V*_m_) were calculated within the pCLAMP software offline (Sims et al., 2014; Brennan et al., 2015; Brennan et al., 2023; Brennan et al., 2024).

#### 2.7.4 Whole-cell currents

In voltage-clamp mode, the membrane potential was held at 0 mV to inactivate most voltage-gated currents to record the metabolically sensitive outward K_ATP_ current. To record different ionic currents in male- and female-derived cardiomyocytes, a voltage step protocol from a holding potential of – 70 mV was used as outlined in **Supplementary Figure 1**. This allowed recordings of the inward rectifier current (− 100 to − 40 mV), calcium current (− 50 to 40 mV), and the delayed rectifier currents (− 50 to 40 mV).

### 2.8 Video edge detection measurements of contraction

Cells were allowed to adhere to a glass coverslip mounted in a Dagan HW-30 heated perfusion chamber (Dagan Corp, USA) for 10 min prior to experimentation. Solutions were perfused at a rate of 5 ml/min and at 32 °C ± 2 °C. Cardiomyocytes were stimulated to contract using electric field stimulation (EFS) at 1 Hz. Contractile function was observed via a JVC closed-circuit television (CCTV) camera and recorded to a digital video disc (DVD) for offline analysis. Video edge detection was carried out using a video-edge detection system (Crescent Electronics, Salt Lake City, Utah, USA), digitised using a Minidigi 1B interface (Axon Instruments), and recorded using Axoscope 10.7 software (Axon Instruments, RRID: SCR_011323). Arbitrary measurement units were calibrated, using an eyepiece graticule, to micrometres.

### 2.9 Metabolic inhibition and washout protocol

Freshly isolated ventricular cardiomyocytes were stimulated to contract using electric field stimulation (EFS) at 1 Hz. Contractile function was observed via a JVC CCTV camera and recorded to DVD for offline analysis. Cardiomyocytes were perfused (5 ml/min at 32 °C–34 °C) with NT solution for 3 min and substrate-free metabolic inhibition Tyrode’s solution (SFT-MI; containing 2 mM cyanide [Merck, Gillingham, UK] and 1 mmol/L iodoacetic acid [Merck, Gillingham, UK]) for 7 min, followed by 10 min of NT (reperfusion).

In this assay, cells are exposed to 7 min of SFT-MI solution. As the perfusion with the SFT-MI progresses, [ATP]_i_ is depleted, leading to opening of the metabolically sensitive Kir6.2/SUR2A channels. This causes contractile failure due to a large hyperpolarising K^+^ current. As metabolic inhibition continues, intracellular Ca^2+^ ([Ca^2+^]_i_) rises due to depleted [ATP]_i_, no longer able to maintain [Ca^2+^]_i_ homeostasis, and the cell slowly enters a rigor contracture. On washout, in cells where [Ca^2+^]_i_ has elevated significantly (around 30% of cells), hypercontracture occurs, where these cells then fragment, losing membrane integrity, and so label with Trypan Blue. A subset of cells does not undergo a damaging hypercontracture, where some will partially recover their [Ca^2+^]_i_ but are not contractile, often referred to as stunned, and a final group that recovers contractile function (contractile recovery). The proportion of cells that recover their contractile function and label with Trypan Blue is a marker of cardioprotection; an increased recovery and survival in this assay is a marker of protection, whilst a decrease is a marker of cardiotoxicity. Furthermore, a delay in the time to contractile failure is indicative of a delay in ATP depletion and is also considered a marker of cardioprotection.

Analysis was performed on the video file recordings to measure contractile and morphological changes of cardiomyocytes, such as contractile recovery after simulated ischaemia, time to contractile failure, and cell survival as measured by Trypan Blue exclusion (Sims et al., 2014; Brennan et al., 2015; Brennan et al., 2019; Brennan et al., 2022; Brennan et al., 2023; Brennan et al., 2024). At least two repeats per day were recorded for each condition, and the data were expressed as the mean of the day, *n* = animals, for statistical analysis.

### 2.10 Fura-2 measurements of intracellular calcium transients

Isolated cardiomyocytes were incubated with 5 µM Fura-2-AM or Fluo-4-AM (Thermo Fisher, Altrincham, UK) indicator at room temperature for 30 min. Cells were allowed to adhere to a glass coverslip mounted in a Dagan HW-30 heated perfusion chamber (Dagan Corp, USA) for 10 min prior to experimentation. Solutions were perfused at a rate of 5 ml/min and at 32 °C ± 2 °C. Cardiomyocytes were stimulated to contract using EFS at 1 Hz. For longer measurements of the Ca^2+^ response to metabolic inhibition, Fura-2 fluorescence was excited at 340 and 380 nm excitation light from a PTI-monochromator, with emissions collected above 520 nm using an Andor Zyla 4.5 camera, with images recorded to WinFluor software (John Dempster, University of Strathclyde, Glasgow, UK). Images were recorded once every 3 s throughout the 20-min protocol, with the exception of short periods of rapid recording to measure the Ca^2+^ transients. To record the Ca^2+^ transients at the start of the recording, at the end of metabolic inhibition, and at the end of washout, images were acquired at a rate of 25 frames per second, and 2 pixel × 2 pixel binning was used to improve the signal-to-noise ratio given the short exposure time (20 ms). The mean Fura-2 ratio for each cell was taken as the baseline (diastolic) Fura-2 ratio between transients. To measure changes in transient amplitude or changes in fluorescence over time with metabolic inhibition, the time-point (Fura-2) ratio was measured, and the baseline was subtracted to give a change in ratio value.

For detailed measurements of Ca^2+^ transients in **Supplementary Figure 1**, Fluo-4 fluorescence data were acquired at ~ 50 frames per second with fluorescence excited at 480 nm and an exposure time of 20 ms. Fluorescence was normalised to basal (the mean of the diastolic fluorescence), giving *F*/*F*_0_ values. For each cell within a field of view, the peak fluorescence, the duration of the transient, and the area under the curve were calculated using Graphpad Prism 10. The mean of 10 calcium transients was calculated for each cell, and the data were expressed as the mean of the day.

### 2.11 Data and statistical analysis

All statistical analyses were performed in GraphPad Prism 10 (GraphPad Software Inc., La Jolla, CA, USA, RRID: SCR_002798); the statistical tests used for each dataset are reported in each figure legend. Student’s paired or unpaired *t-*test, nonparametric Mann–Whitney *U* test, one-way or two-way ANOVA with posttests were performed as indicated in the figure legends. Statistics were carried out using *n* = animal, unless otherwise stated in the figure legends. Data are plotted as bar charts with individual mean values plotted. Standard deviation error bars are shown. Indicative colour-coded ranges have been shown in the figures to indicate the normal, cardiotoxic, and cardioprotective ranges of behaviour in our different assays. The ranges were determined from our extensive use of these protocols (Sims et al., 2014; Brennan et al., 2015; Brennan et al., 2019; Brennan et al., 2022; Brennan et al., 2023; Brennan et al., 2024; Esposito et al., 2025).

### 2.12 Materials

#### 2.12.1 Drugs

Adenosine (Merck, Gillingham, UK), cyanide (Merck, Gillingham, UK), iodoacetic acid (Merck, Gillingham, UK), PNU37883A (Biotechne Ltd., Abingdon, UK), and pinacidil (Merck, Gillingham, UK) were used.

## 3 Results

### 3.1 The open probability of Kir6.1 channels is increased in ventricular cardiomyocytes isolated from an ischaemic preconditioned heart

Kir6.1 channels are functionally expressed at the cell surface in ventricular myocytes (Brennan et al., 2024) and, unlike the canonical cardiac Kir6.2/SUR2A channel complex, are constitutively active under normal physiological conditions, suggesting less reliance on ATP for their modulation. To assess the effects of cardioprotective stimuli on Kir6.1 activity, whole hearts underwent a well-established ischaemic preconditioning (IPC) protocol that involved stopping perfusion for 5-min intervals with 5 min of reperfusion in between these cycles (Rodrigo and Samani, 2008; Sims et al., 2014; Brennan et al., 2015). Kir6.1 activity was measured in the cell-attached patch recording configuration from single cells isolated from these hearts, with channel openings detected as deflections of ~ 5.5 pA from baseline corresponding to a single-channel conductance of 40 pS, as previously described (Brennan et al., 2024). Examination of the Kir6.1 open probability, with adjustments for the number of channels in the patch (NP_O_), in cardiomyocytes isolated from control or IPC hearts showed that it was not normally distributed. In cell-attached patch recording, the mean Kir6.1 NP_O_ was significantly increased from ~ 0.11 in control cells to ~ 0.46 in IPC cells (**Figures 1A, B**). Furthermore, the distribution of NP_O_ was shifted compared with control cells, with ~ 81% of cells being in the group with an NP_O_ greater than 0.16 (**Figure 1C**). The mean Kir6.1 NP_O_ was similarly different when the mean by animal was expressed. Given the wide variability of NP_O_ recorded, the geometric mean of the day was used to create each data point to reduce the influence of “outliers” on the mean (**Figure 1D**).

Exposure of control and IPC cardiomyocytes to a metabolic inhibition solution, a SFT-MI containing 2 mmol/L cyanide and 1 mmol/L iodoacetic acid, and widely used in previous studies (Sims et al., 2014; Brennan et al., 2015; Brennan et al., 2019; Brennan et al., 2022; Brennan et al., 2023), caused substantive opening of Kir6.2/SUR2A channels (70 pS) with a mean time to opening of ~ 145 s (**Figures 1E, F**). Plotting the time to Kir6.2/SUR2A channel activation within a cell against the Kir6.1 NP_O_ of that cell prior to metabolic inhibition reveals a correlation. Cells with a higher Kir6.1 NP_O_ in resting conditions take longer to activate Kir6.2/SUR2A channels during metabolic inhibition (**Figure 1G**).

In action potential recordings, the action potential duration to 90% repolarised (APD_90_) was significantly shorter, and the resting membrane potential was more negative in IPC cardiomyocytes compared with control cells (**Figures 1H–J**). Furthermore, on treatment with 3 µM PNU37883A (PNU), a Kir6.1 pore-blocking compound that is selective for Kir6.1 at 3 µM (Kovalev et al., 2004; Brennan et al., 2024), the APD_90_ shortening and hyperpolarisation of IPC cardiomyocytes were reversed (**Figures 1K–M**), consistent with a role for Kir6.1 in modulating the action potential and membrane potential. Finally, the Kir6.1 NP_O_ was significantly reduced in both control and IPC cardiomyocytes with PNU treatment (**Figure 1N**).

### 3.2 Pharmacological inhibition of Kir6.1 reverses cardioprotection in IPC cardiomyocytes

The effect of Kir6.1 upregulation was assessed in a metabolic inhibition and washout experiment that has been widely used to assess cardioprotection and toxicity (Lawrence et al., 2001; Rodrigo et al., 2002; Rodrigo et al., 2004; Collins and Rodrigo, 2010; Sims et al., 2014; Brennan et al., 2015; Brennan et al., 2019; Brennan et al., 2022; Brennan et al., 2023). To assess the Ca^2+^ handling properties, or contractile function, and the ability to survive a metabolic insult, cells were exposed to 7 min of perfusion with a metabolic inhibition solution that causes ATP depletion and therefore calcium accumulation. On washout with NT solution, cells either enter a state of hypercontracture, which can be detected with Trypan Blue (identified as dead), retain their structural integrity but remain noncontractile (stunned), or recover their contractile function.

In this study, it was hypothesised that potentiation of Kir6.1-containing channel activity by IPC would impart a shortened Ca^2+^ transient duration, together with a reduced Ca^2+^ transient amplitude, to reduce the accumulation of cytoplasmic Ca^2+^ ([Ca^2+^]_i_) during each contractile cycle. Such a reduction in [Ca^2+^]_i_ accumulation would mean less ATP consumption to restore homeostasis, and so, during ischaemia, where ATP synthesis is arrested, a slower depletion of intracellular [ATP]. To assess this, cardiomyocytes were loaded with Fura2-AM and exposed to the metabolic inhibition and washout protocol outlined in the methods.

**Figure 2:**
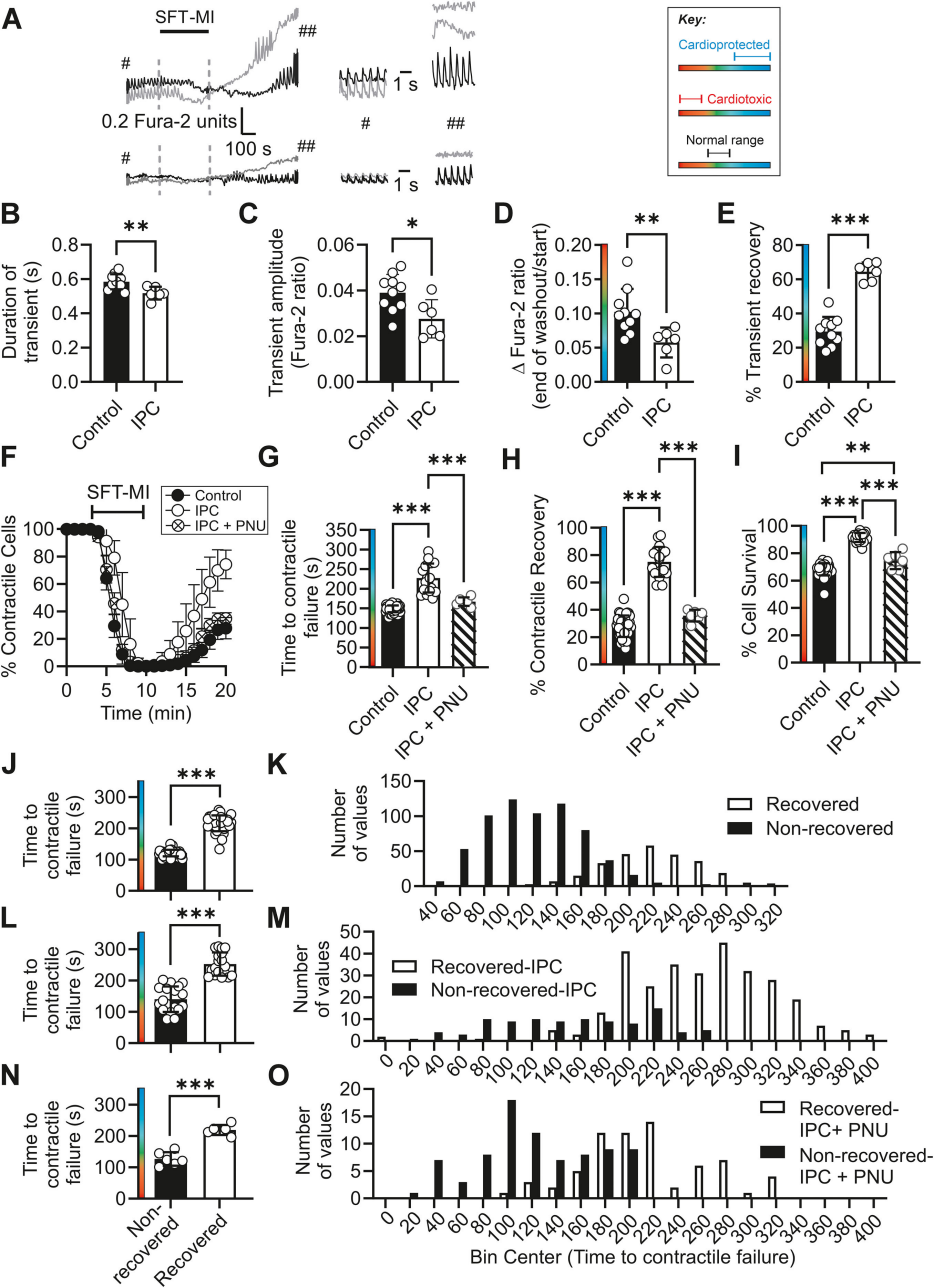
Ischaemic preconditioning of the whole heart imparts a cardioprotected phenotype to isolated cardiomyocytes that can be blocked by Kir6.1 inhibition with PNU. **(A)** Example traces of Fura-2 ratio from recovered (black line) and nonrecovered (grey line) cardiomyocytes in control (top traces) and IPC cardiomyocytes (bottom traces). Example Ca^2+^ transients at the start of the recording (^#^) and at the end (^##^) of the washout. **(B)** Mean duration of the Ca^2+^ transients at the start of the protocol prior to metabolic inhibition (^**^p = 0.009, unpaired *t*-test). **(C)** Mean amplitude of the Ca^2+^ transients at the start of the protocol prior to metabolic inhibition (^*^p = 0.018, unpaired *t*-test). **(D)** Mean change in diastolic Fura-2 ratio at the end of the protocol (^**^p = 0.006, unpaired *t*-test). **(E)** Mean transient recovery in control and IPC cardiomyocytes (^***^p < 0.001, unpaired *t*-test). For **(A–E)**, *n* = 10 and 6 animals for control and IPC cardiomyocytes, respectively. **(F)** Time course showing the percentage of contractile cells during a metabolic inhibition and washout protocol, in which cells are perfused for 3 min with NT, followed by 7 min with SFT-MI solution, which is then washed out for 10 min. Control, IPC, and IPC cells treated with 3 µmol/L PNU37883A (PNU) are compared. **(G)** Mean time to contractile failure (^***^p < 0.001), **(H)** mean percentage of cells recovering contractile function (^***^p < 0.001), and **(I)** mean percentage of cells surviving the protocol (as identified by Trypan Blue labelling) (^**^p = 0.005; ^***^p < 0.001). For all groups, one-way ANOVA with Tukey’s posttest was used (*n* = 39, 18, and 9 for control, IPC, and IPC with PNU, respectively). **(J)** Mean time to contractile failure in control cells, separated into cells that did (216.1 s ± 25.3 s) and did not (120.8 s ± 10.6 s) recover contractile function (^***^p < 0.001, unpaired *t*-test, *n* = 39 animals). **(K)** Histogram showing the distribution of time to contractile failure in control cells, separated into cells that recovered their contractile function (white bars, 271 cells) and those that did not (black bars, 650 cells) from 39 animals. **(L)** Mean time to contractile failure for all IPC data collected in this study, separated into cells that did (252.3 s l± 37.2 s) and did not (140.4 s ± 40.7 s) recover contractile function (^***^p < 0.001, unpaired *t*-test, *n* = 18 animals). **(M)** Histogram showing the distribution of time to contractile failure, separated into cells that recovered their contractile function (white bars, 295 cells) and those that did not (black bars, 97 cells) from 18 animals. **(N)** Mean time to contractile failure for all PNU-treated IPC cell data, separated into cells that did (218.9 s ± 15.9 s) and did not (126.7 s ± 21.7 s) recover contractile function (^***^p < 0.001, unpaired *t*-test, *n* = 6 animals). **(O)** Histogram showing the distribution of time to contractile failure, separated into cells that recovered their contractile function (white bars, 69 cells) and those that did not (black bars, 82 cells) from six animals.

In cardiomyocytes isolated from an IPC heart, there was a significant shortening of transient duration prior to metabolic inhibition, together with a significantly smaller transient amplitude compared with cardiomyocytes isolated from a control heart (**Figures 2A–C**). In control heart cardiomyocytes, there was a significantly larger [Ca^2+^]_i_ accumulation at the end of the protocol compared with the beginning, indicative of an increased number of cardiomyocytes losing structural integrity during the protocol compared with IPC heart-derived cells. This was highlighted by the increased number of IPC cells, ~ 70% compared with ~ 30% in control cells, able to recover Ca^2+^ transients at the end of the protocol (**Figures 2D, E**).

When assessing contractile function, IPC heart-derived cardiomyocytes showed a significantly delayed time to contractile failure and an increase in percentage contractile recovery and cell survival when compared with control cardiomyocytes (**Figures 2F, G**). Consistent with the hypothesis that increased Kir6.1 channel activity was imparting protection, selective inhibition of Kir6.1 with PNU37883A reversed the protection seen in IPC heart-derived cardiomyocytes so that they were indistinguishable from control heart-derived myocytes (**Figures 2F–H**). An exception to this was percentage cell survival, which remained significantly improved (**Figure 2I**). On further analysis of the time to contractile failure, the mean time, when split into those cells that did and did not recover contractile function, showed a significant difference. Cells that failed to recover contractile function had an earlier mean failure time compared with those that recovered contractile function in control heart-derived cardiomyocytes (**Figure 2J**). These data are also presented in a distribution bar chart showing the distribution of times to contractile failure by those cells that did and did not recover contractile function (**Figure 2K**). Compared with the cells that had undergone the IPC protocol, the mean time to contractile failure for each group did not substantially change mean times to contractile failure for each group had not substantially changed (compared **Figures 2L, J**). The distribution of cells between the recovered and nonrecovered groups shifts (compared **Figures 2M, K**). In IPC cardiomyocytes, this shift toward a delayed time to contractile failure was reversed when treated with PNU (**Figures 2N, O**). These data suggest that the time to contractile failure during metabolic inhibition is linked to the fate of the cell following washout; the earlier a cell enters a state of contractile failure, presumably via ATP depletion triggering opening of the Kir6.2/SUR2A complex, the less likely the cell is to recover contractile function by the end of the 10-min washout period. Cardioprotection with IPC significantly shifts the cells toward a delayed time to contractile failure and increased contractile recovery.

**Figure 3:**
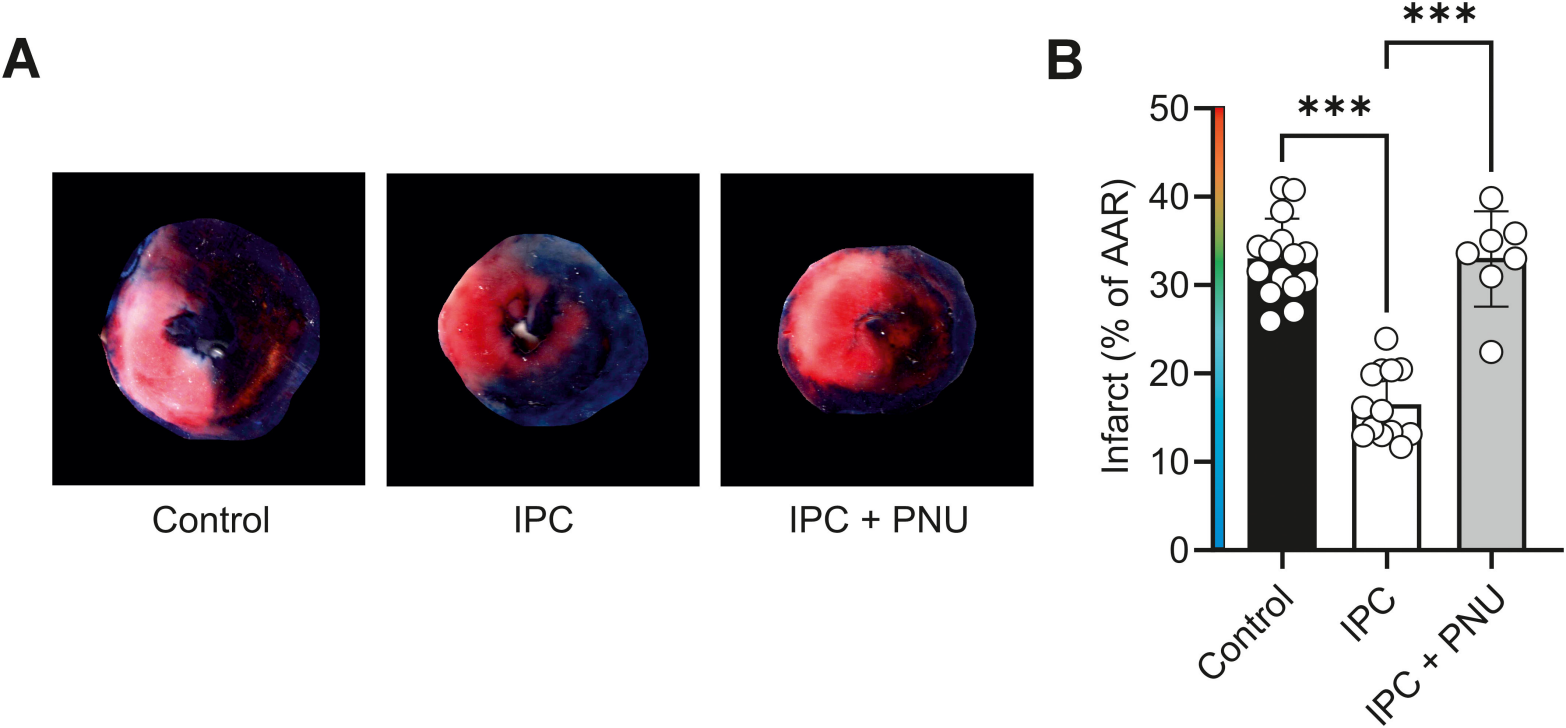
Ischaemic preconditioning of the whole heart imparts cardioprotection that can be reversed by selective Kir6.1 inhibition with PNU37883A. **(A)** Example myocardial slices from control, IPC, and IPC + PNU-treated hearts. **(B)** Mean infarct expressed as a percentage of the area at risk. ^***^p < 0.0001. One-way ANOVA with Tukey’s posttest was used (*n* = 15, 13, and 7 for control, IPC, and IPC + PNU-treated hearts, respectively).

The cardioprotection against infarct imparted by an IPC protocol in a whole-heart *ex vivo* left anterior descending coronary ligation protocol was also assessed. In hearts undergoing an IPC protocol, the infarct size was significantly reduced. Treatment with 3 µM PNU to selectively block Kir6.1 channels abolished the protection afforded by the IPC protocol in the *ex vivo* heart (**Figure 3**).

**Figure 4:**
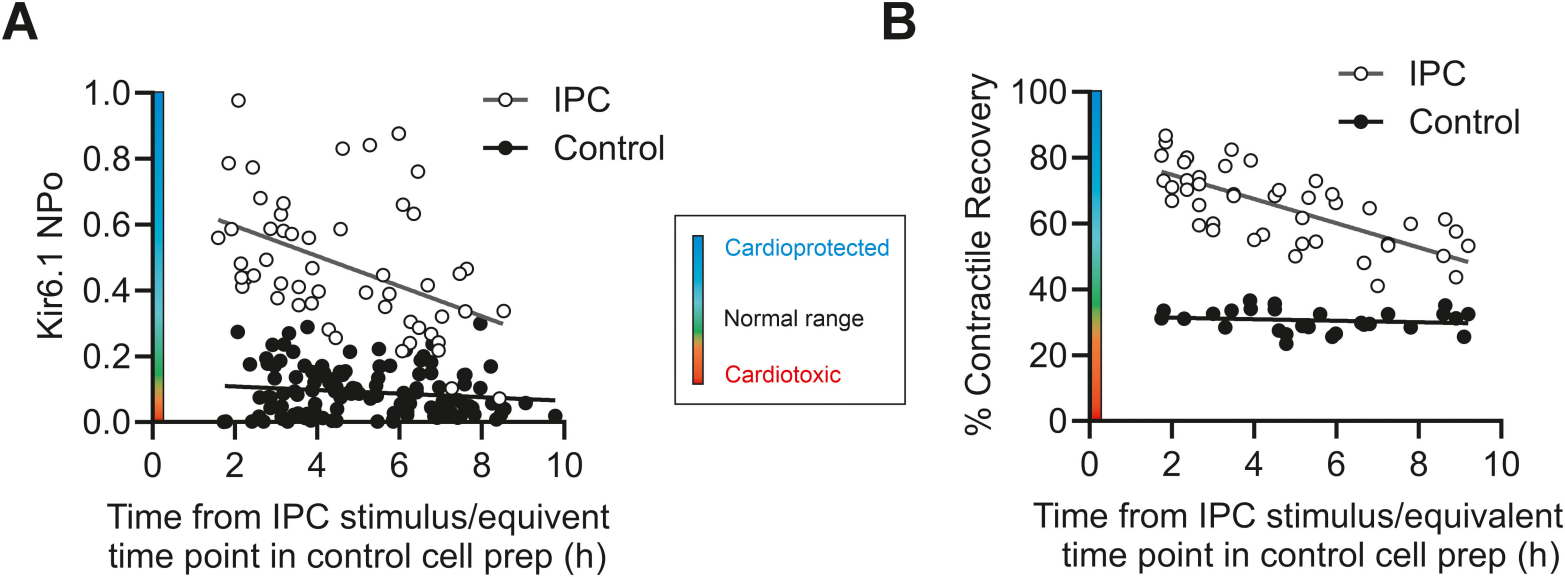
IPC-induced increase in NP_O_ and increase in contractile recovery wanes after 8 h following the IPC stimuli. **(A)** Kir6.1 NP_O_ plotted against the time at which the data were recorded from the end of the whole heart IPC protocol prior to cell isolation, or an equivalent time in a control heart. Data show a trend toward a lower Kir6.1 NP_O_ in IPC cells by ~ 8 h (*n* = 66 and 122 IPC vs. control cells). **(B)** Percentage contractile recovery plotted against the time at which the data were recorded from the end of the whole heart IPC protocol prior to cell isolation, or an equivalent time in a control heart. Data show a trend toward a lower percentage contractile recovery by ~ 8 h (*n* = 45 IPC and 28 control experiments from 18 animals in each group).

The phenomenon of cardioprotection is typically believed to occur over two time periods: a first window of protection that lasts for up to 8 h, and a second window of protection that lasts between 24 and 48 h (Hausenloy and Yellon, 2010). To investigate whether the cardioprotection observed in this study was time-dependent, the Kir6.1 NP_O_ and percentage contractile recovery in IPC and control cardiomyocytes were plotted against the time from the IPC stimulus (or the equivalent time point in control cells) (**Figure 4**). In IPC cardiomyocytes, there was a trend toward higher NP_O_ and higher contractile recovery the closer, temporally, to the IPC stimulus. These data correlate with the known loss of protection seen as the first window of IPC wanes by ~ 8 h.

The findings in (**Figures 1**-**4**) suggest that the open probability of Kir6.1 channels and the degree of cardioprotection are linked. The higher the Kir6.1 NP_O_ in cells, the more delayed the time to Kir6.2/SUR2A channel opening and the time to contractile failure. High Kir6.1 NP_O_ cells also show increased contractile recovery and survival following metabolic stress. These are all hallmarks of a cardioprotected phenotype. This is further replicated in the whole heart, where an IPC protocol protects the myocardium from infarction, whereas selective block of Kir6.1 channels with PNU increases infarct size.

### 3.3 In control cardiomyocytes, pharmacological activation of Kir6.1 channel activity is cardioprotective, whereas inhibition is cardiotoxic

Having established that an increased Kir6.1 channel activity is seen in IPC cardiomyocytes and that the cardioprotection afforded by IPC could be abolished by selective Kir6.1 blockade, our next experiments were designed to assess whether increasing Kir6.1 NP_O_ pharmacologically is cardioprotective and whether Kir6.1 inhibition is cardiotoxic in cells isolated from normally perfused hearts.

**Figure 5:**
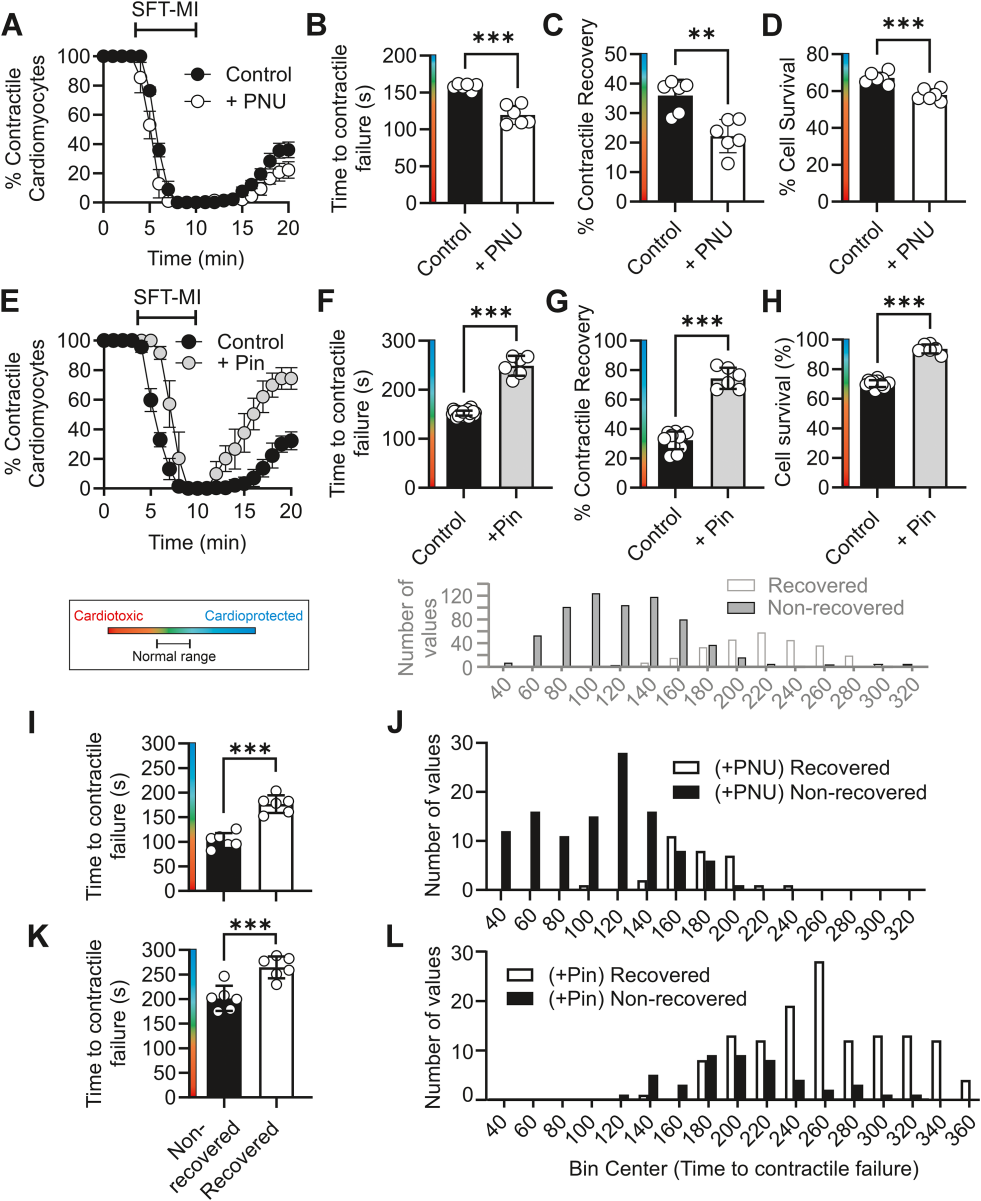
Direct pharmacological inhibition of Kir6.1 is cardiotoxic, whereas direct pharmacological activation of Kir6.1 is cardioprotective. **(A)** Time course showing the effects of PNU37783A on the percentage of contractile cardiomyocytes throughout the metabolic inhibition and washout protocol. **(B)** Mean time to contractile failure (^***^p < 0.001), **(C)** mean percentage of cells recovering contractile function (^**^p = 0.002), and **(D)** mean percentage of cells surviving the protocol (as identified by Trypan Blue labelling) (^***^p < 0.001). For control and PNU-treated groups, unpaired *t*-test was used (*n* = 6 animals for each group). **(E)** Time course showing the effects of pinacidil on the percentage of contractile cardiomyocytes throughout the metabolic inhibition and washout protocol. **(F)** Mean time to contractile failure (^***^p < 0.001), **(G)** mean percentage of cells recovering contractile function (^***^p < 0.001), and **(H)** mean percentage of cells surviving the protocol (as identified by Trypan Blue labelling) (^***^p < 0.001). For control and pinacidil-treated groups, unpaired *t*-test was used (*n* = 23 and 6 animals for control and pinacidil-treated groups, respectively). **(I)** Mean time to contractile failure in PNU-treated cells, separated into cells that did (176.7 s ± 18.05 s) and did not (102.5 s ± 15.2 s) recover contractile function (^***^p < 0.001, unpaired *t*-test, *n* = 6 animals). **(J)** Histogram showing the distribution of time to contractile failure separated into cells that recovered their contractile function (white bars, 31 cells) and those that did not (black bars, 113 cells) from six animals. **(K)** Mean time to contractile failure in pinacidil-treated cells, separated into cells that did (264.6 s ± 22.2 s) and did not (201.5 s ± 22.2 s) recover contractile function (^***^p < 0.001, unpaired *t*-test, *n* = 6 animals). **(L)** Histogram showing the distribution of time to contractile failure, separated into cells that recovered their contractile function (white bars, 135 cells) and those that did not (black bars, 46 cells) from six animals. Control data from **Figure 2** are plotted in grey above **(J, L)** to allow direct comparison with the distributions of contractile recovery in control cells.

In the metabolic inhibition and washout protocol on control cardiomyocytes, inclusion of the Kir6.1 pore blocker PNU37883A in control cells caused a shortening in the time to contractile failure, a reduction in the percentage of cells recovering contractile function on washout, and a decrease in the percentage cell survival as assessed by Trypan Blue exclusion (**Figures 5A–D**), all hallmarks of cardiotoxicity in this assay. Selective potentiation of Kir6.1 with 50 µM pinacidil, a concentration that does not significantly activate Kir6.2-containing channels (Brennan et al., 2024), caused a substantial delay in the time to contractile failure, an increase in the percentage of contractile recovery, and cell survival, markers of cardioprotection in this assay (**Figures 5E–H**). As previously stated, the time to contractile failure in the cells in this assay is linked to the depletion of ATP and the opening of the metabolically sensitive Kir6.2 channel (Brennan et al., 2015). Further analysis of the time to contractile failure showed that the mean time, when split into those cells that did and did not recover contractile function, differed significantly. Those that failed to recover showed a significantly earlier mean time to failure compared with those that recovered (**Figures 5I–L**). These findings were exacerbated in PNU-treated cells, where significantly more cells were in the “nonrecovered” group (**Figures 5I, J**), whereas in the pinacidil-treated cells, significantly more cells were in the “recovered” group (**Figures 5K, L**). These findings suggest that in control cells, increasing Kir6.1 activity delays contractile failure (presumably by ATP preservation), whereas inhibiting Kir6.1 channels accelerates contractile failure.

**Figure 6:**
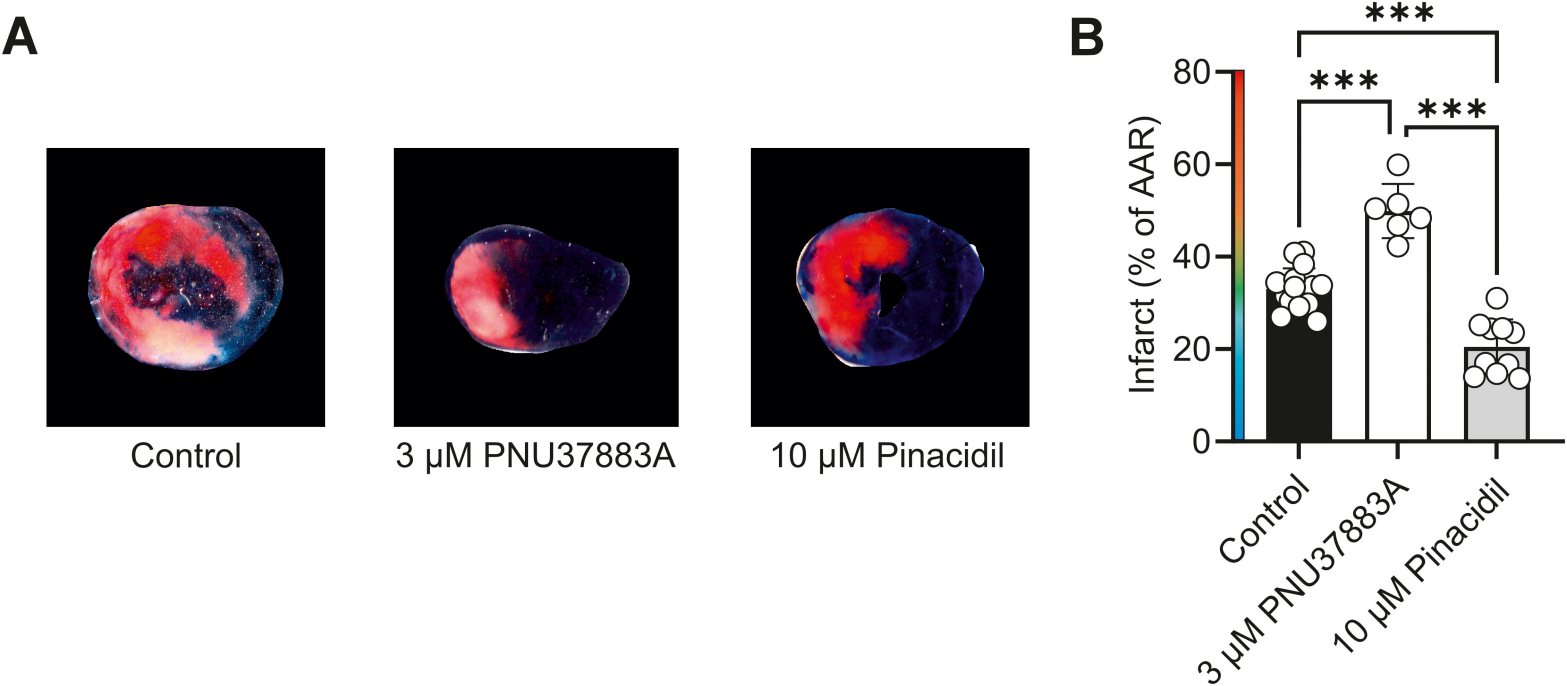
Infarct size in an *ex vivo* whole heart coronary ligation protocol is increased by selective Kir6.1 inhibition with PNU37883A and reduced by selective Kir6.1 activation with pinacidil. **(A)** Example images of slices of myocardium labelled with area at risk and area of necrosis. **(B)** Mean infarct size expressed as a percentage of the area at risk in control, PNU, or pinacidil treatment groups. ^***^p < 0.001. One-way ANOVA with Tukey’s posttest was used (*n* = 15, 6, and 10 hearts in control, PNU, or pinacidil-treated groups, respectively).

In an *ex vivo* whole-heart left anterior descending coronary artery ligation protocol, 3 µmol/L PNU37883A increased infarct size, whereas 10 µmol/L pinacidil reduced infarct size, further suggesting that increasing Kir6.1 activity was protective in these experiments (**Figure 6**).

These data suggest that direct Kir6.1 channel manipulation affects the level of protection afforded to cardiomyocytes, with an increase in Kir6.1 NP_O_ being protective and a decrease being toxic.

### 3.4 Adenosine, a physiological mediator of IPC, increases NP_O_ and imparts cardioprotection to control cardiomyocytes

**Figure 7:**
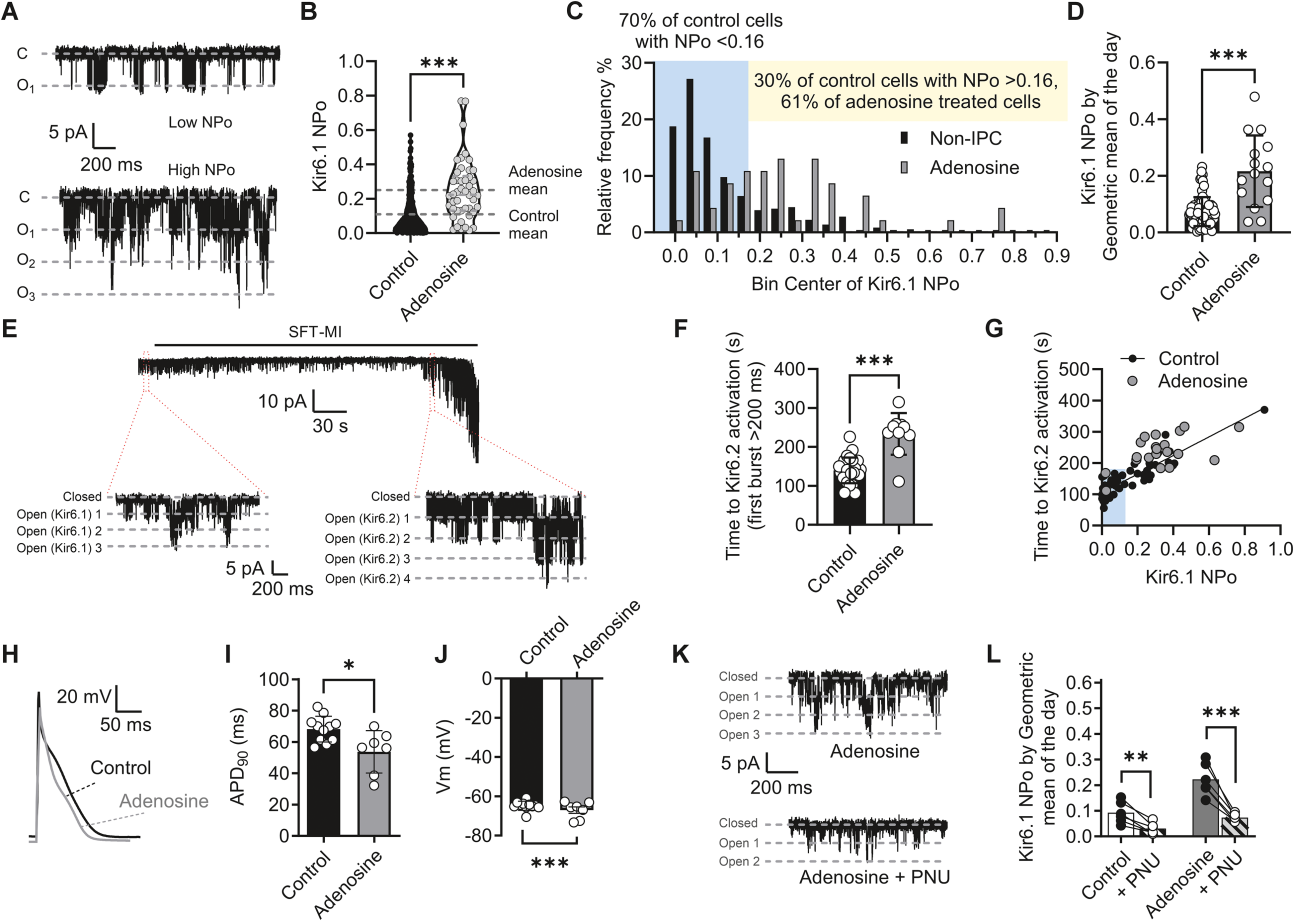
Pretreatment with 100 µmol/L adenosine increases Kir6.1 NP_O_ and delays the time to Kir6.2/SUR2A channel activation by metabolic inhibition. **(A)** Example traces of Kir6.1 activity from an adenosine-treated cell with a low NP_O_ and one with a high NP_O_. **(B)** Mean NP_O_ from 357 control (0.11 ± 0.10) and 46 adenosine-treated cells (0.25 ± 0.17). ^***^p < 0.001. Data were not normally distributed, and therefore a nonparametric Mann–Whitney *U* test was used. **(C)** Distribution histogram of NP_O_ for adenosine-treated and nontreated cells in 0.05 bins. In nontreated cardiomyocytes, 70% of cells had an NP_O_ < 0.16, whereas 30% had an NP_O_ > 0.16. In adenosine-treated cardiomyocytes, this was shifted to 61% of cells having an NP_O_ > 0.16. **(D)** Mean Kir6.1 NP_O_ calculated as the mean by animal (from the geometric mean of the day). ^***^p < 0.001. Data were not normally distributed, and therefore a nonparametric Mann–Whitney *U* test was used. **(E)** Example trace of a cell-attached patch recording from an adenosine-treated cardiomyocyte showing the opening of Kir6.2/SUR2A following perfusion with SFT-MI solution. Expanded traces show Kir6.1 openings prior to and Kir6.2/SUR2A openings during SFT-MI perfusion. **(F)** Mean time to Kir6.2/SUR2A channel opening in nontreated and adenosine-treated cells, showing a significant delay in the time to Kir6.2/SUR2A opening (*p* < 0.001, unpaired *t*-test, *n* = 26 and 10 animals in the nontreated and adenosine-treated groups, respectively). **(G)** Time to Kir6.2/SUR2A opening during SFT-MI perfusion plotted against the Kir6.1 NP_O_ of that cell (*n* = 57 [26 animals] and 23 [10 animals] control vs. adenosine-treated cells, respectively). **(H)** Example action potential traces control and adenosine-treated cardiomyocytes. **(I)** Mean APD_90_ and **(J)** membrane potential in control and adenosine-treated cardiomyocytes (^*^p = 0.030, ^**^p < 0.01, unpaired *t*-test, *n* = 11 and 7 animals in the control and adenosine-treated groups, respectively). **(K)** Example traces of Kir6.1 NP_O_ in the absence and presence of 3 µM PNU37883A. **(L)** Mean NP_O_ in the absence and presence of 3 µM PNU37883A in control and adenosine-treated cardiomyocytes (^**^p = 0.006, ^***^p < 0.001, *n* = 6 [9] and 6 [10] animals [cells]). Repeated-measures two-way ANOVA with uncorrected Fisher’s posttest was used.

The mechanisms by which cardioprotection is imparted to cardiomyocytes *in vivo* are not fully understood. Reports suggest that local release of adenosine as a metabolic waste product during ischaemia may be part of that process, and addition of adenosine as a pretreatment has been shown to be cardioprotective (Miura and Tsuchida, 1999; Takahama et al., 2009). To assess whether an increased Kir6.1 NP_O_ would be imparted by pretreatment with adenosine, cardiomyocytes isolated from a control heart were perfused for 5 min with 100 µM adenosine. Following treatment, the adenosine perfusate was switched back to NT solution, and cell-attached patch recordings were made (**Figures 7A–D**). Adenosine as a pretreatment was able to increase the NP_O_ from ~ 0.11 to ~ 0.25 (**Figure 7B**) and also demonstrated a shift in the number of cells showing an NP_O_ greater than 0.16 to 61% from 30% in the nontreated cells (**Figure 7C**), with a significant increase in mean Kir6.1 NP_O_ by animal (**Figure 7D**). Consistent with our IPC data, there was also a delay in the time to Kir6.2/SUR2A channel opening following exposure to metabolic inhibition solution (**Figures 7E, F**) that also correlated with the increased NP_O_ in the cell-attached patch data recorded from adenosine-pretreated cells (**Figure 7G**).

In action potential recordings, the APD_90_ was significantly shorter and the resting membrane potential more negative in adenosine-treated cardiomyocytes compared with control cells (**Figures 7H–J**). Finally, the Kir6.1 NP_O_ was significantly reduced in both control and adenosine-treated cardiomyocytes with PNU treatment (**Figures 7K, L**).

**Figure 8:**
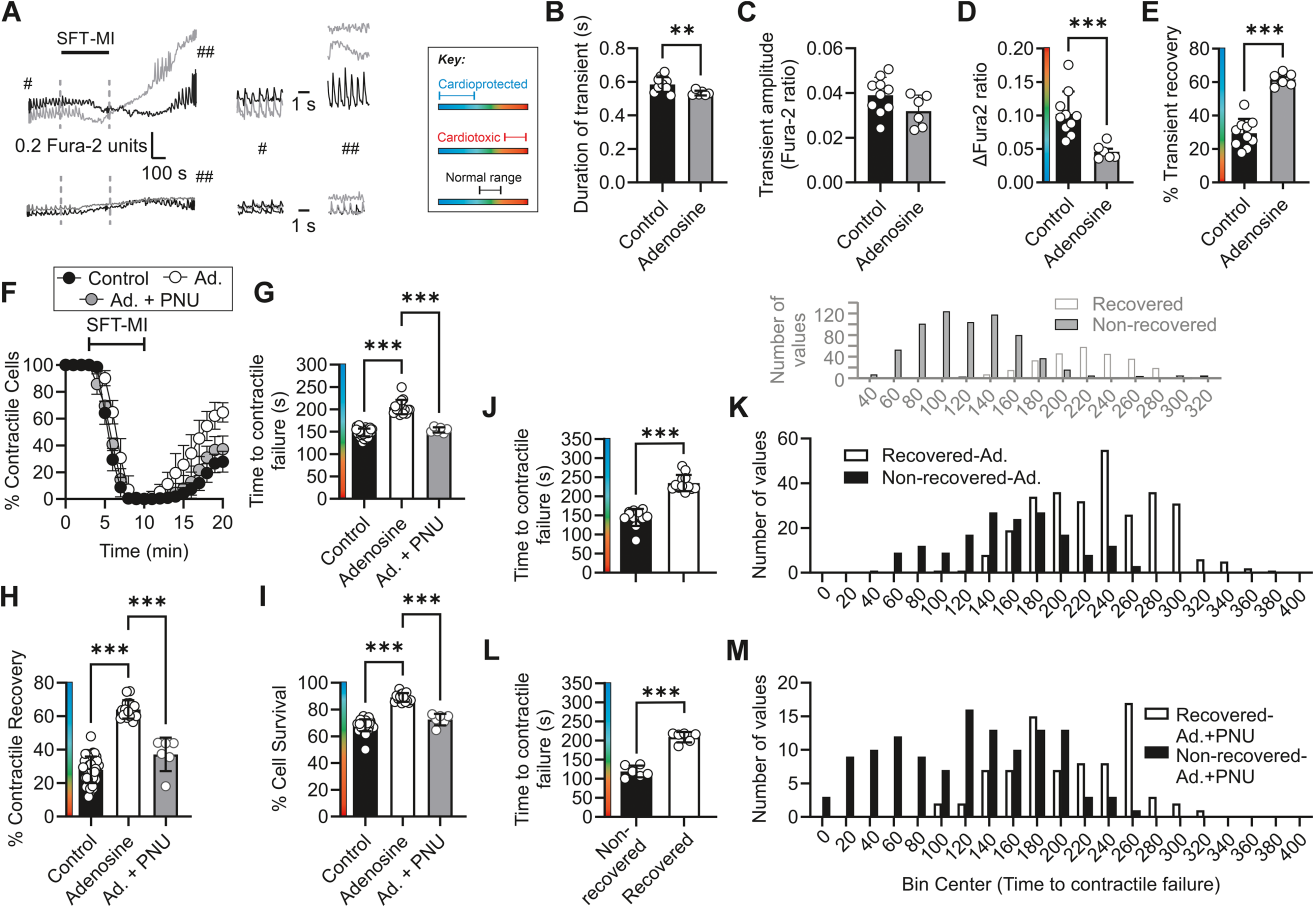
Pretreatment with 100 µmol/L adenosine imparts cardioprotection to cardiomyocytes, which is abolished by selective Kir6.1 block with 3 µmol/L PNU. **(A)** Example traces of Fura-2 ratio from recovered (black line) and nonrecovered (grey line) cardiomyocytes in control (top traces) and adenosine-treated (bottom traces). Example Ca^2+^ transients from the start of the recording (^#^) and at the end (^##^) of the washout. **(B)** Mean duration of the Ca^2+^ transients at the start of the protocol prior to metabolic inhibition (^**^p = 0.005, unpaired *t*-test). **(C)** Mean amplitude of the Ca^2+^ transients at the start of the protocol prior to metabolic inhibition (*p* = 0.095, unpaired *t*-test). **(D)** Mean change in diastolic Fura-2 ratio at the end of the protocol (^***^p = 0.001, unpaired *t*-test). **(E)** Mean transient recovery in control, IPC, and adenosine-treated cardiomyocytes (^***^p < 0.001, unpaired *t*-test). For **(A–E)**, *n* = 10 and 6 animals for control and adenosine-treated cells, respectively. **(F)** Time course showing the effects of adenosine pretreatment on the percentage of contractile cardiomyocytes throughout the metabolic inhibition and washout protocol. **(G)** Mean time to contractile failure (^***^p < 0.001), **(H)** mean percentage of cells recovering contractile function (^***^p < 0.001), and **(I)** mean percentage of cells surviving the protocol (as identified by Trypan Blue labelling) (^***^p < 0.001). For nontreated, adenosine pretreated, and adenosine pretreated with PNU-treated groups, one-way ANOVA with Tukey’s posttest was used (*n* = 39, 15, and 6 animals). **(J)** Mean time to contractile failure for adenosine-treated cells, separated into cells that did (234.5 s ± 21.2 s) and did not (144.5 s ± 22.2 s) recover contractile function (^***^p < 0.001, unpaired *t*-test, *n* = 12 animals). **(K)** Histogram showing the distribution of time to contractile failure, separated into cells that recovered their contractile function (white bars, 293 cells) and those that did not (black bars, 166 cells) from 12 animals. **(L)** Mean time to contractile failure in adenosine- and PNU-treated cells, separated into cells that did (208.7 s ± 13.7 s) and did not (118.7 s ± 15.3 s) recover contractile function (^***^p < 0.001, unpaired *t*-test, *n* = 6 animals). **(M)** Histogram showing the distribution of time to contractile failure, separated into cells that recovered their contractile function (white bars, 79 cells) and those that did not (black bars, 122 cells) from six animals. Control data from **Figure 2** are plotted in grey above **(K, M)** to allow direct comparison with the distributions of contractile recovery in control cells.

In adenosine-treated cardiomyocytes, there was a significant shortening of Ca^2+^ transient duration prior to metabolic inhibition, together with a significantly smaller transient amplitude compared with nontreated control cardiomyocytes (**Figures 8A–C**). Similar to IPC cardiomyocytes, adenosine-treated cardiomyocytes showed reduced [Ca^2+^]_i_ accumulation and increased transient recovery following the metabolic inhibition and washout protocol compared with control (nontreated) cells (**Figures 8D, E**).

Consistent with the findings in IPC cardiomyocytes, adenosine pretreatment delayed the time to contractile failure and increased the contractile recovery and cell survival of cardiomyocytes (**Figures 8F–I**). Additionally, inhibition of the Kir6.1 channel by PNU reversed the cardioprotection afforded by adenosine pretreatment so that the PNU-treated cells were indistinguishable from the untreated control cardiomyocytes (**Figures 8F–I**).

Adenosine also delayed the time to contractile failure in a larger proportion of cardiomyocytes in metabolic inhibition, indicative of a protective effect. Again, selective inhibition of Kir6.1 with PNU shortened the time to contractile failure and also reduced the number of cells recovering their contractile function (**Figures 8J–M**). These findings were consistent with the hypothesis that an increased Kir6.1 NP_O_ provided reduced ATP consumption and so delayed the depletion of intracellular ATP during metabolic inhibition.

### 3.5 Female cardiomyocytes and whole hearts are inherently cardioprotected compared with male-derived cardiomyocytes and have a higher Kir6.1 NP_O_

It has long been established that premenopausal women are intrinsically cardioprotected compared with age-matched men. This protection wanes postmenopause, where men and women equalise in their risk of cardiovascular events (Yang and Reckelhoff, 2011; Barrett-Connor, 2013; Murphy and Steenbergen, 2014; Blenck et al., 2016).

**Figure 9:**
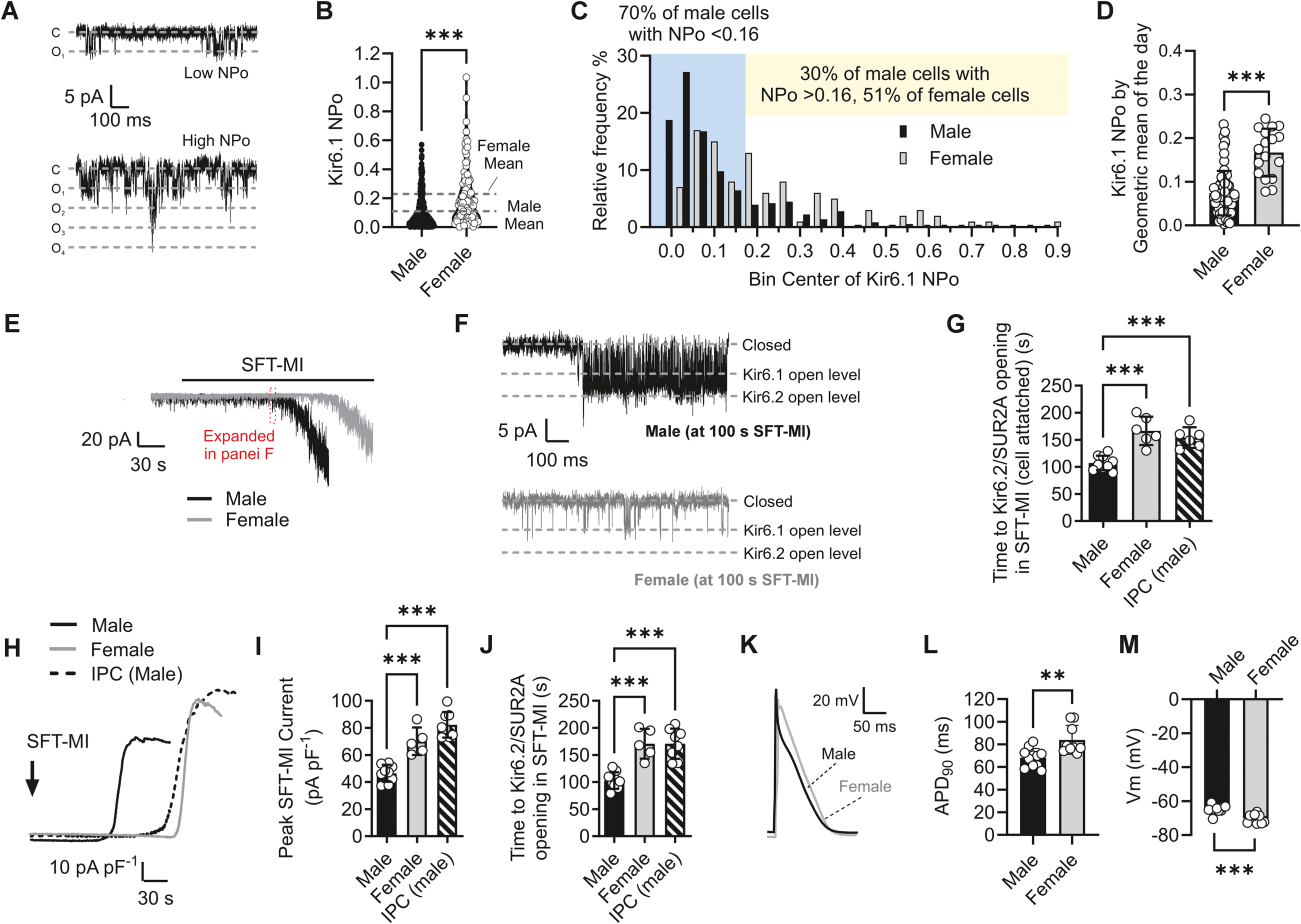
Female cardiomyocytes have a higher Kir6.1 NP_O_ than age-matched male ones. **(A)** Example traces of Kir6.1 activity from a female heart-derived cardiomyocyte with a low NP_O_ and one with a high NP_O_. **(B)** Mean NP_O_ from 357 male heart-derived cardiomyocytes (0.11 ± 0.10) and 61 female heart-derived cardiomyocytes (0.22 ± 0.16). Data were not normally distributed, and therefore a nonparametric Mann–Whitney *U* test was used. **(C)** Distribution histogram of NP_O_ for female and male heart-derived cardiomyocytes in 0.05 bins. In male heart-derived cardiomyocytes, 70% of cells had an NP_O_ < 0.16, whereas 30% had an NP_O_ > 0.16. In female heart-derived cardiomyocytes, this was shifted to 46% of cells having an NP_O_ > 0.16. **(D)** Mean Kir6.1 NP_O_ in male and female cells plotted by animal. Each data point represents the geometric mean of NP_O_ recordings from that animal. Data were not normally distributed; therefore, a nonparametric Mann–Whitney *U* test was used, *n* = 73 and 12 animals, male and female, respectively. **(E)** Example traces of cell-attached recordings in male- and female-derived cardiomyocytes. **(F)** Example expanded traces from the time point in **(E)**, indicated with a dotted red box, showing Kir6.2/SUR2A activity in male-, but not in female-derived cardiomyocytes, following 100 s of perfusion with metabolic inhibition solution. **(G)** Mean times to the first burst of Kir6.2/SUR2A activity that was longer than 200 ms (^***^p < 0.001, one-way ANOVA with Tukey’s posttest, *n* = 9, 6, and 6 animals [male, female, and male-IPC, respectively]). **(H)** Example traces from a male (black line) and female (grey line) heart-derived cardiomyocyte of a metabolic inhibition-activated whole-cell current primarily composed of K_ATP_ current. **(I)** Mean peak amplitude in male, female, and male-IPC cardiomyocytes (^***^p < 0.001). **(J)** Mean time to metabolically sensitive current activation, defined as the time from the start of metabolic inhibition to a current increase of 25 pA over the basal current (^***^p < 0.001, one-way ANOVA with Tukey’s posttest, *n* = 9, 6, and 8 animals, male, female, and male-IPC, respectively). **(K)** Example action potentials from male- and female-derived cardiomyocytes. **(L)** Mean APD_90_ (^**^p = 0.003) and **(M)** membrane potential (^***^p < 0.001) in male- and female-derived cardiomyocytes (unpaired *t*-test, *n* = 11 male and 9 female animals).

In cell-attached patch recordings, there was a significant increase in Kir6.1 NP_O_ in female-derived (0.22) compared to male-derived (0.11) cardiomyocytes, with 51% of cells having an NP_O_ greater than 0.16, compared with 30% in male cells (**Figures 9A–D**).

Consistent with male-derived IPC and adenosine-treated cardiomyocytes, female-derived cardiomyocytes with no cardioprotective intervention showed a delayed time to metabolically sensitive Kir6.2/SUR2A channel opening in both cell-attached patch recordings (**Figures 9E–G**) and whole-cell recordings (**Figures 9H–J**). In these experiments, female cardiomyocytes that had not undergone any protective protocol and male IPC heart-derived cells were indistinguishable. Interestingly, there have been reports of K_ATP_ channel activity being linked with cardioprotection in female cells (Chicco et al., 2007; Edwards et al., 2009). Indeed, total K_ATP_ current density, as measured by SFT-MI-induced activation of metabolically sensitive K_ATP_ currents in whole-cell recordings, was significantly larger in female and male-IPC cardiomyocytes compared with control male cells (**Figures 9H, I**). Finally, in action potential recordings, female cells showed a significantly *longer* APD_90_ than male cells. However, there was a more hyperpolarised membrane potential, consistent with an increased Kir6.1 activity (**Figures 9 K–M**).

**Figure 10:**
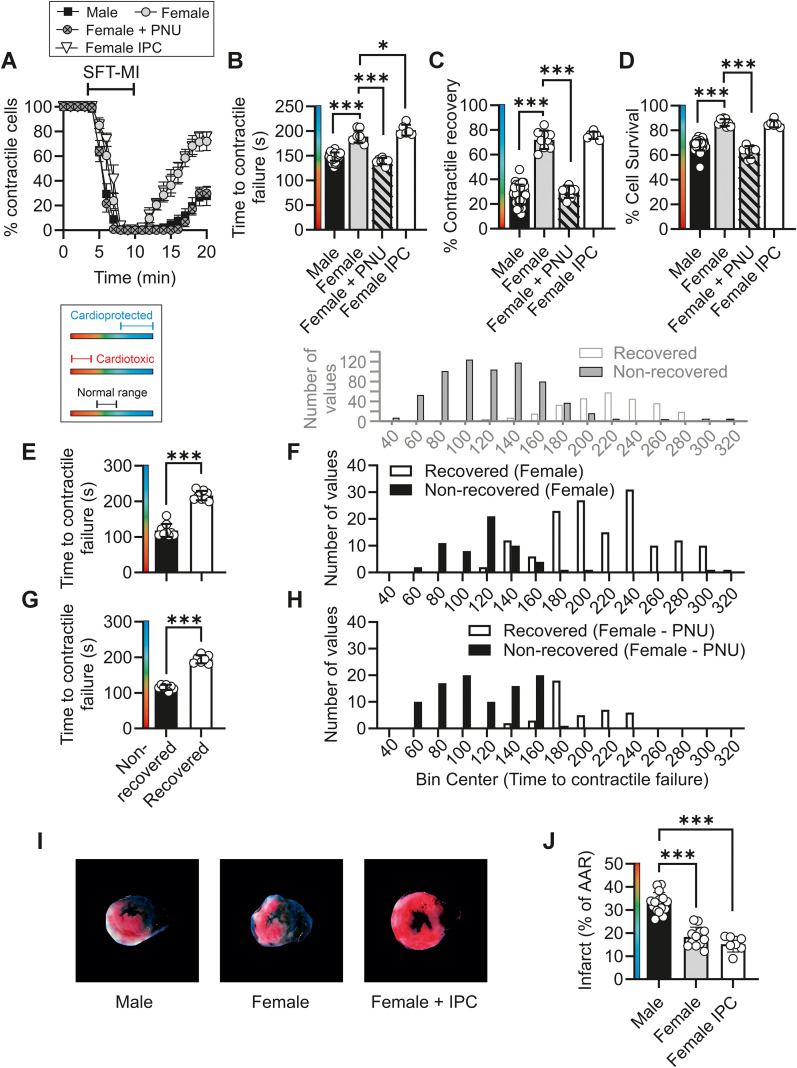
Female cardiomyocytes are inherently cardioprotected and have a higher Kir6.1 NP_O_ than age-matched males. **(A)** Time course showing the effects of sex on the percentage of contractile cardiomyocytes throughout the metabolic inhibition and washout protocol. **(B)** Mean time to contractile failure, **(C)** mean percentage of cells recovering contractile function, and **(D)** mean percentage of cells surviving the protocol (as identified by Trypan Blue labelling). For each parameter measured, there was a significant difference between male and female heart-derived cardiomyocytes and female heart-derived cardiomyocytes with 3 µmol/L PNU (^*^p = 0.02, ^***^p < 0.001, one-way ANOVA with Tukey’s posttest, *n* = 39 male, 9 female animals, 7 female with PNU, and 6 female IPC). **(E)** Mean time to contractile failure, separated into cells that did (216.4 s ± 13.3 s) and did not (118.2 s ± 18.4 s) recover contractile function (^***^p < 0.001, unpaired *t*-test, *n* = 9 animals). **(F)** Histogram showing the distribution of time to contractile failure, separated into cells that recovered their contractile function (white bars, 149 cells) and those that did not (black bars, 56 cells) from nine animals. **(G)** Mean time to contractile failure, separated into cells that did (195.2 s ± 27.1 s) and did not (115.1 s ± 34.32 s) recover contractile function (^***^p < 0.001, unpaired *t*-test, *n* = 7 animals). **(H)** Histogram showing the distribution of time to contractile failure, separated into cells that recovered their contractile function (white bars, 41 cells) and those that did not (black bars, 94 cells) from seven animals. Data from male control cells in **Figure 2** are shown above **(F, H)** for direct comparison. **(I)** Example images of myocardial slices from male, female, and female IPC hearts following an *ex vivo* coronary ligation protocol. **(J)** Mean percentage infarct of the area at risk in male, female, and female IPC hearts (^***^p < 0.001, one-way ANOVA with Tukey’s posttest, *n* = 12 male, 9 female, and 7 female IPC hearts).

Assessment of cardioprotection, using the metabolic inhibition and washout protocol, also demonstrated that female-derived cardiomyocytes had a cardioprotected phenotype that was blocked by treatment with PNU (**Figures 10A–D**). Ischaemic preconditioning did not further improve the outcomes for the female cardiomyocytes (**Figures 10A–D**). As with other protective treatments, the time to contractile failure data, when separated into cells that did and did not recover their contractile function, demonstrated that more female cells were in the “recovered” group and had a delayed time to contractile failure. This protection was reversed by treatment with PNU (**Figures 10E–H**).

Finally, in the whole heart coronary ligation model, female hearts showed a smaller infarct size compared with age-matched male hearts (**Figures 10I, J**).

## 4 Discussion

**Figure 11:**
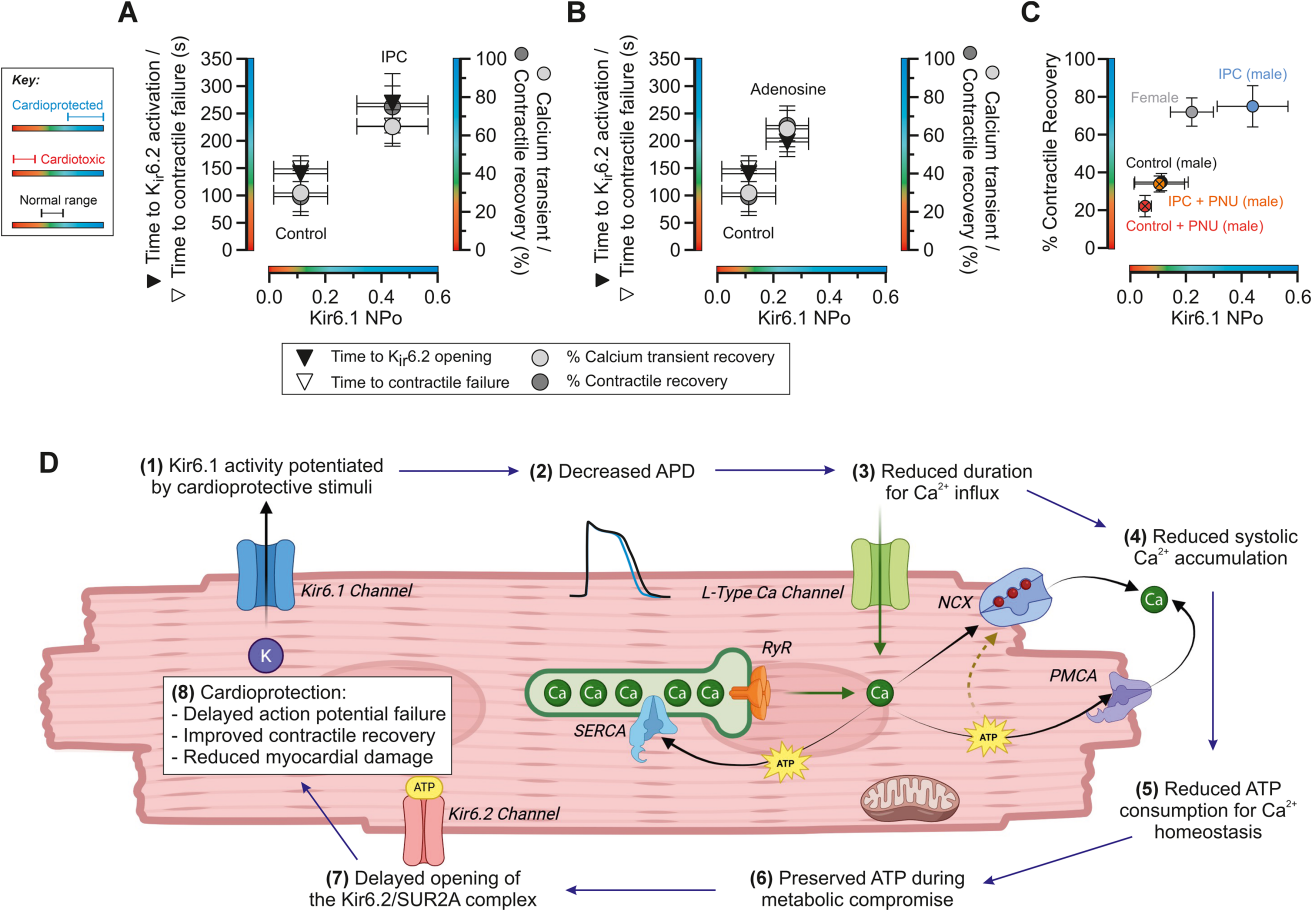
Summary of the main findings. **(A)** Summary of the main differences between control cells and IPC cardiomyocytes. Time to Kir6.2 activation, contractile failure (left *y*-axis), and percentages of calcium transient recovery and contractile recovery (right *y*-axis) against the mean Kir6.1 NP_O_ for each group. A higher NP_O_, higher contractile and transient recovery, delayed time to contractile failure, and Kir6.2 channel activation are all being markers of cardioprotection; IPC cardiomyocytes are in the upper right quadrant of the plot, compared with control in the lower left. **(B)** As in **(A)**, but plotted for adenosine pretreated cells, showing a shift toward cardioprotection, although less pronounced than the gold standard of ischaemic preconditioning. **(C)** Summary of the proposed link between mean contractile recovery following the metabolic inhibition and washout protocol and mean Kir6.1 NP_O_ in male, female, male IPC, and PNU-treated cells. **(D)** Cartoon representing the proposed mechanism of protection in cardiomyocytes (1). Kir6.1 is potentiated, which leads to shortening of APD (2). This reduces the duration of opening of the l-type calcium channels (3), which reduces the calcium accumulation in the cytoplasm (4). This, in turn, reduces the ATP consumption required to remove the calcium (5), thereby preserving intracellular ATP levels for a longer period. This allows the cell to maintain its ATP and calcium homeostasis for a longer period during a metabolic insult (6). This delays the time of the Kir6.2/SUR2A complex opening (7) and imparts cardioprotection (8), manifesting as delayed AP failure, improved contractile recovery, and reduced myocardial damage. Panel C partially created in BioRender. Rainbow, R. (2026) https://BioRender.com/7ky2s0z.

In this study, we provide evidence for a link between an increased ventricular sarcolemmal Kir6.1 NP_O_ and cardioprotection. The link between K_ATP_ channel pharmacology and cardioprotection has been widely reported; the link to the specific channel subunits involved has not. In this study, we show that the mean NP_O_ of the Kir6.1 channel is increased in two different cardioprotective stimuli, IPC and pretreatment with adenosine. In both cases, this increased NP_O_ is coupled with an increase in time required for Kir6.2/SUR2A channels to open, indicative of a delay in the time to ATP depletion. There is also a delay in the time to contractile failure, which is associated with an increase in Kir6.2/SUR2A activity. There is also an increase in the number of cells recovering their Ca^2+^ transients, recovering their contractile function, and an increase in cell survival following a metabolic inhibition and washout protocol. These findings are consistent with our previous study, in which we demonstrated a delay in Kir6.2/SUR2A opening in different models of cardioprotection (Brennan et al., 2015). Our findings in this and our previous studies are summarised in **Figures 11A, B**.

We have previously demonstrated two distinct populations of K_ATP_ channels in the heart with different pore subunits that exhibit distinct biophysical and pharmacological properties, with the constitutively active component being absent in *kcnj8^−/−^* knockout animals (Brennan et al., 2024). Furthermore, we found no evidence of heteromultimerisation of the pore-forming subunits. A dimeric fusion of Kir6.1 and Kir6.2 showed intermediate conductance compared to homotetramers of Kir6.1 and Kir6.2 (Brennan et al., 2024), consistent with the findings of other groups (Babenko et al., 2000; Cui et al., 2001). We propose that there are two distinct components to the cardiac K_ATP_ current: a low-conductance, constitutively active Kir6.1-containing current and a higher-conductance, metabolically sensitive Kir6.2-containing current, each of which has different roles in cardioprotection. Kir6.1 fine-tunes the resting membrane potential and action potential duration to control Ca^2+^ influx in normal physiological conditions and during the early stages of metabolic compromise. The Kir6.2 component, however, responds to the catastrophic fall in ATP concentrations seen in sustained ischaemia, passing a large hyperpolarising K^+^ current that causes contractile failure to preserve cellular integrity.

Forty years after Noma’s first description of K_ATP_ in the heart (Noma, 1983), we still do not fully understand the role of these channels, despite their known property of matching electrical excitability to cellular metabolism. In recent years, Kir6.1 channels have been shown to regulate heart rate by altering the sinoatrial node APD (Aziz et al., 2018) and to promote coronary vasodilation (Aziz et al., 2014) due to the channel’s expression in vascular smooth muscle. Here, we report a fundamental role for the Kir6.1 channel in the heart at the ventricular sarcolemmal surface, where potentiation of the channel confers protection against simulated ischaemia. This supports previous findings from multiple laboratories that have identified Kir6.1 channel expression on the membrane surface (Singh et al., 2003; Morrissey et al., 2005a; Morrissey et al., 2005b). This report does not rule out or support the existence of Kir6.1 channels in the mitochondria; however, it supports a role for a sarcolemmal Kir6.1 channel in physiological/pathophysiological conditions and in ischaemic protection.

The data in this study suggest an important link between the Kir6.1 NP_O_ and the response of cells to metabolic inhibition. Contractile recovery, as a marker of cardioprotection, is worse in control cardiomyocytes with selective Kir6.1 inhibition with PNU (**Figure 11C**). Contractile recovery is increased in ischaemic preconditioned cardiomyocytes with an increased Kir6.1 NP_O_ but is reversed with selective Kir6.1 inhibition (**Figure 11C**). In female cardiomyocytes, the findings suggest that the intrinsic cardioprotection observed in premenopausal women may, in part, be due to an increase in Kir6.1 NP_O_ (**Figure 11C**). The intrinsic protection in females is likely to be multifactorial, given that oestrogen-dependent cardioprotection can modulate long-term expression of signalling factors and ion channels that may improve the metabolic resistance of the cell to ischaemia. It is compelling that Kir6.1 activity is increased in females without cardioprotective intervention, that Kir6.1 channel block reduces the protected status, and that ischaemic preconditioning does not further reduce infarct size, suggesting at least some link between Kir6.1 channels and intrinsic cardioprotection in females. Furthermore, both the contractile and calcium transient amplitude, and the durations of both the contractile and calcium response to electric field stimulation in female cells are significantly smaller than in male cardiomyocytes (**Supplementary Figures 1A–H**). In keeping with reports in the literature, we did not find any significant changes in inward rectifier or calcium currents between male and female cardiomyocytes, but we did observe a significantly smaller IK current in female cardiomyocytes (**Supplementary Figures 1I–L**). This reduced IK current correlates with a longer action potential duration in female cells (Wasserstrom et al., 2008; Parks and Howlett, 2013). These findings suggest a less clear link with the Kir6.1 channel and the calcium handling in female cells, given the different morphology of the action potential. Despite this, the inhibition of Kir6.1 with PNU did worsen the outcome in a metabolic inhibition and washout experiment (**Figures 10A–D**), suggesting that it does play some role in protection. This is an area that warrants further investigation, including in postmenopausal women, to assess whether Kir6.1 stimulation can restore protection when the protective influence of cycling oestrogen is diminished.

Our data show that, under control conditions, a majority of cells have a Kir6.1 NP_O_ lower than 0.16 (70%). This correlates well with our data investigating time to contractile failure, where we observe that, under control conditions, 30% of cells recover their contractile function, with those cells showing a significantly delayed time to contractile failure. This correlates with time to Kir6.2 opening, which we suggest is a marker of ATP depletion, being delayed in cells with a higher Kir6.1 NP_O_. Our data suggest that there is a shift in the Kir6.1 NP_O_ toward a higher level of opening (> 0.16) in IPC cardiomyocytes (81%), adenosine-treated cells (61%), or in female heart-derived cells (46%), which results in delayed contractile failure and improved contractile recovery. These data suggest an important link between the Kir6.1 NP_O_ and the level of protection afforded to the cell.

Further evidence suggesting a link between the K_ATP_ channel family and cardioprotection comes from our assessment of the time to contractile failure, a surrogate marker for ATP depletion. In our analysis of the time to contractile failure, we demonstrate that the earlier a cell enters contractile failure, indicative of Kir6.2/SUR2A activation by depleted intracellular ATP, the more likely this cell is to fail to recover or to undergo hypercontractility, leading to cell death. Our analysis shows that the times to failure between the group of cells that recovered contractile function and those that did not were remarkably consistent across the different treatments. Our data show that it was the proportion of cells in the different groups that was altered, where, following cardioprotective stimuli, more cells failed to contract later and were therefore able to recover contractile function more readily. This correlates with our previous findings of delayed Kir6.2/SUR2A activation, delayed calcium increases, delayed ATP depletion, and delayed mitochondrial depolarisation following cardioprotective stimuli (Brennan et al., 2015). Furthermore, we now demonstrate that female cells show increased Kir6.1 NP_O_, together with a cardioprotected phenotype.

The prevalence of coronary heart disease in postmenopausal women on hormone replacement therapies (HRT) was reduced compared to women not taking HRT in epidemiological studies (Stampfer et al., 1985). Such findings led to the hypothesis that HRT given to those women over the age of 70 and at risk of coronary heart disease or stroke may reduce the risk. In the early 2000s, the Women’s Health Initiative (WHI) launched a number of intervention trials to investigate whether there was a benefit to treating with HRT, all of which showed a paradoxical worsening of prognosis for those on either oestrogen/progesterone or solely oestrogen therapy, resulting in an early termination of the studies (Fletcher and Colditz, 2002; Rossouw et al., 2002; Grodstein et al., 2003; Herrington and Howard, 2003; Wassertheil-Smoller et al., 2003; Hulley and Grady, 2004; Heiss et al., 2008). Given that all cardioprotected processes used in this study showed an increased Kir6.1 NP_O_, direct targeting of Kir6.1 could be an alternative mechanism to promote cardioprotection in both men and women at risk of coronary heart disease. Our group has previously reported a similar cardioprotective effect of potentiation of another cardiac potassium current, IKs, which led to a shortened APD and reduced ATP consumption through a mechanism similar to that outlined in this study (Brennan et al., 2023).

A limitation of this study was that experiments were performed on isolated cardiomyocytes or on whole hearts *ex vivo*. PNU37883A, thought to be a selective pore blocker of Kir6.1 (Cui et al., 2003; Kovalev et al., 2004; Teramoto, 2006), has also been suggested to have additional off-target effects, such as a diuretic effect in the kidney (Humphrey, 2006); however, at the significantly lower concentration used in this study (3 µM), the effects would be Kir6.1-specific (Kovalev et al., 2004; Brennan et al., 2024). It remains to be investigated whether Kir6.1 channels confer protection against ischaemia *in vivo*. Further complexity is added by the numerous reports identifying both pro- and antiarrhythmic effects of K_ATP_ channel openers and blockers (Brady and Terzic, 1998). During ischaemia, significant shortening of the APD caused by K_ATP_ channel activation can result in two contrasting consequences (1): reduced Ca^2+^ influx via voltage-gated Ca^2+^ channels, leading to decreased arrhythmia risk from delayed afterdepolarizations, and (2) reduced refractoriness, resulting in an increased occurrence of re-entrant arrhythmias (Brady and Terzic, 1998). This report raises the possibility that perhaps only the canonical of the two sarco-K_ATP_ channels in cardiomyocytes (Kir6.2/SUR2A), rather than all K_ATP_ channels, is mechanistically responsible for the reported arrhythmias during ischaemia. This is a reasonable suggestion given the distinct properties of the two K_ATP_ channels at the ventricular membrane. Kir6.2 channels, activated at high concentrations of pinacidil or metabolic poison, consequently cause a marked shortening of the APD. This is comparable to the established effects of gain-of-function hERG mutations in short-QT, which cause arrhythmias via shortening of the APD and refractoriness (Brugada et al., 2004). In contrast, Kir6.1 channels are constitutively active and provide a subtle shortening of the action potential and so may not affect refractoriness. IPC cardiomyocytes, with the highest Kir6.1 NP_O_, showed a modest action potential shortening in our hands compared with a substantive shortening seen in cells that are treated with pinacidil, which also opens the Kir6.2/SUR2A channel. These findings suggest that Kir6.1 activation may have a “ceiling” effect on the APD and so limit the propensity for proarrhythmic behaviour. Development of a potential therapeutic targeting Kir6.1 would, therefore, require significant care to ensure that it is as selective as possible for the Kir6.1 isoform.

Interestingly, key differences between global and vascular-specific Kir6.1 knockout could be explained by ventricular Kir6.1 expression (Miki et al., 2002; Aziz et al., 2014; Dart, 2014). Both global and vascular-specific Kir6.1-KO mice are hypertensive, but global Kir6.1 knockouts also display ST elevation, indicative of acute myocardial ischaemia, and are prone to sudden death (Miki et al., 2002). The lack of ST elevation and sudden death in vascular-specific Kir6.1 knockouts could be attributed to ventricular Kir6.1 imparting crucial protection to the myocardium (Aziz et al., 2014).

Ultimately, a role for this Kir6.1 channel is supported by our current report and brings a large body of literature together. A greater understanding of the role of Kir6.1 in all areas of the myocardium, in physiological and ischaemic conditions, will be required to determine whether selective Kir6.1 modulation could be a novel therapeutic target for the treatment of acute coronary syndromes and/or an overlooked target in cardiac safety screens. These findings of a constitutively active Kir6.1 channel in the sarcolemmal membrane of ventricular myocytes pose an important consideration for the development of novel compounds by the pharmaceutical industry; should Kir6.1 and Kir6.2 be included in cardiac panel safety screens, such as the Comprehensive *in vitro* Proarrhythmia Assay (CiPA) cardiac safety initiative? Pharmacological blockade of the Kir6.1 current, in our hands, had a toxic effect of shortening the time to contractile failure, reducing contractile recovery, and increasing cell death in a metabolic inhibition and washout protocol, and also increasing infarct size in a whole heart *ex vivo* model. These findings suggest that pharmacological blockade of Kir6.1 by compounds such as rosiglitazone may help to explain their putative cardiotoxicity, the effect of which on Kir6.1 would not have been detected in current screens.

## 5 Conclusions

This study shows that there are two functionally distinct populations of K_ATP_ channels located at the sarcolemma of cardiomyocytes (1): constitutively active Kir6.1-containing channels that provide initial protection against early-stage metabolic stress, and (2) the canonical Kir6.2/SUR2A channels that activate only after prolonged ischaemia to impart late-stage protection. The finding of a constitutively active Kir6.1-containing population of K_ATP_ channels in cardiomyocytes provides an explanation for the paradox that K_ATP_ channel activation is cardioprotective; however, activation of the known cardiac isoform, Kir6.2/SUR2A, is delayed in cardioprotected cells (Brennan et al., 2015). We present data showing that, in cardiomyocytes, cardioprotective stimuli (e.g., adenosine or IPC) or pharmacological agents increase the constitutive activity of Kir6.1-containing channels, leading to a modest shortening of the APD_90_ and reduced Ca^2+^ influx. This delays mitochondrial depolarisation and ATP depletion, thereby slowing Kir6.2/SUR2A channel activation, which delays AP shortening and contractile failure.

These data suggest that enhanced Kir6.1 activity thus acts as an early-stage protective mechanism that delays the ATP depletion and contractile failure associated with metabolic compromise, thereby preserving Ca^2+^ homeostasis for a longer period, increasing contractile recovery, and decreasing myocardial damage, provided perfusion is restored. Prolonged ischaemia will result in a collapse of intracellular ATP levels and the opening of the canonical Kir6.2/SUR2A channel as an energy-sparing last line of defence.

## Data Availability

The original contributions presented in the study are included in the article/**Supplementary Material**. Further inquiries can be directed to the corresponding author.
