# Supplementary material for: Cardiac ventricular Kir6.1 ATP-sensitive potassium channels: an overlooked effector of cardioprotection
Source: Front Physiol. 2026 Apr 20;17:1808226. doi: 10.3389/fphys.2026.1808226 (PMC13135945; doi:10.3389/fphys.2026.1808226)
Supplement: Supplementary file 1 [file DataSheet1.pdf]

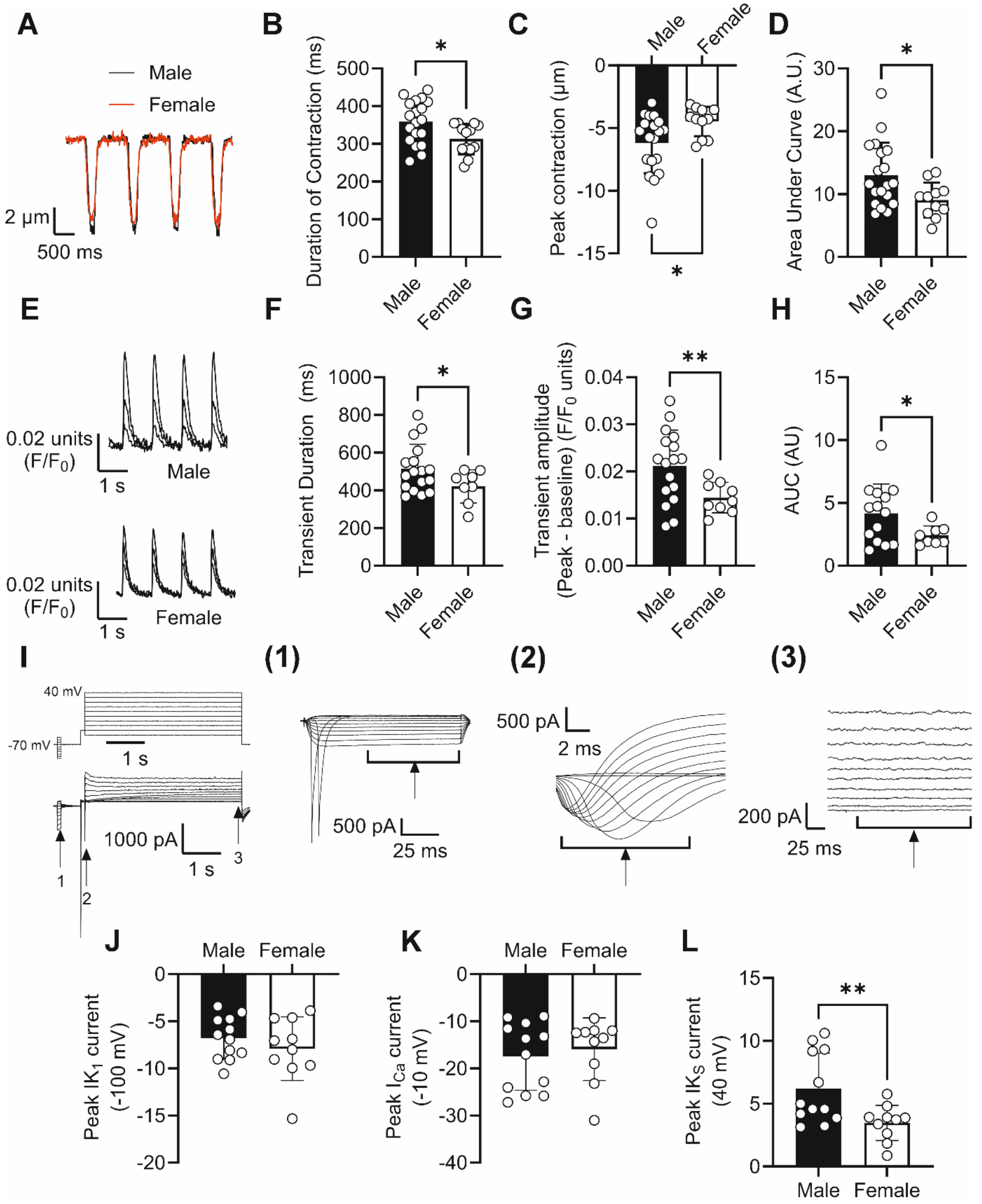

Supplementary figure: Contractile and calcium responses are smaller in female-derived cardiomyocytes

(A) example traces of contraction recorded by video edge detection of male and female-derived cardiomyocyte stimulated to contract at 1 Hz by electric field stimulation. The duration of contraction (*P = 0.017) (B), amplitude of the contraction (P = 0.034) (C), and

the area under the curve (P = 0.025) (D), all showed significant differences between male and female-derived cardiomyocytes (n = 19, 13 animals, male and female respectively, un-paired t-test). (E) example traces of calcium transients, triggered by electric field stimulation, from male and female-derived cardiomyocytes, using Fluo-4. The duration of the calcium transient (P = 0.043) (F), the amplitude of the transient (**P = 0.004) (G), and the area under the curve (*P = 0.018) (H), all showed significant differences between male and female-derived cardiomyocytes (n = 14, 8 animals, male and female respectively, un-paired t-test). (I) Example of a voltage-step protocol in a male cardiomyocyte with expanded section showing (1) IK_1 currents, (2) Ca^{2+} currents and (3) IKs currents. Arrows indicate where mean current, or peak current for each voltage step for Ca^{2+} currents, were taken. Mean data showing peak inward rectifier current at -100 mV (J), peak inward (Ca^{2+} current) at -10 mV (K), and peak outward K current (IKs) at 40 mV (L). There was a significantly smaller IKs current in female cardiomyocytes (*P=0.009, unpaired t-test, 12 male and 10 female animals).
